# Ice nucleation on microcline (001) in the absence of active sites

**DOI:** 10.1038/s41467-026-76548-7

**Published:** 2026-08-25

**Authors:** Florian Schneider, Rasmus Väinö Erik Nilsson, Ralf Bechstein, Hans-Georg Stammler, Bernhard Reischl, Thomas Koop, Angelika Kühnle, Tobias Dickbreder

**Affiliations:** 1 Physical Chemistry I, Faculty of Chemistry, Bielefeld University, Bielefeld, Germany; 2 Institute for Atmospheric and Earth System Research / Physics, Faculty of Science, University of Helsinki, Helsinki, Finland; 3 Anorganische Chemie und Strukturchemie, Faculty of Chemistry, Bielefeld University, Bielefeld, Germany; 4 Atmospheric and Physical Chemistry, Faculty of Chemistry, Bielefeld University, Bielefeld, Germany; 5 Institute of Physical Chemistry, Faculty of Chemistry, University of Vienna, Vienna, Austria

**Keywords:** Surfaces, interfaces and thin films, Atmospheric chemistry

## Abstract

Of the many different ice-nucleating mineral particles, dust from feldspar mineral microcline stands out as being particularly active. However, why microcline outperforms other particles remains a puzzle. Here, we use nanoscale imaging to show that the (001) surface of microcline can induce ice nucleation even in the absence of active sites. In contrast, for the closely related feldspar sanidine, ice nucleation is dominant at step edges as expected. Atomistic simulations suggest that the ice nucleation is induced by a lattice match between the higher-index (10.4) plane of hexagonal ice and microcline. Our findings provide a nanoscopic explanation for microcline’s exceptional ice nucleation ability and demonstrate the importance of taking higher-index surfaces into account.

## Abstract (web summary)

Microcline feldspar exhibits particularly high ice nucleation activity, but the reason for this is still uncertain. Here, the authors show that even the bare microcline terrace, devoid of active sites, exhibits higher ice nucleation activity than related feldspar minerals.

## Introduction

Heterogeneous ice nucleation is at the heart of a wide range of technological and natural fields, ranging from cryopreservation^1^ and the development of anti-icing coatings^2–4^ to the glaciation of clouds^5–11^. The latter process has a profound impact on Earth’s climate as it changes major physical properties, such as the albedo and precipitation efficiency^9,10^. While homogeneous ice nucleation requires temperatures as low as -38 °C^12,13^, heterogeneous ice nucleation in the presence of ice-nucleating particles (INPs) often occurs at significantly higher temperatures^14,15^. Among the great variety of different particles in the atmosphere, mineral dust from microcline—a member of the feldspar group with the chemical formula KAlSi_3_O_8_—is well-known for its exceptional ice-nucleation ability^6,16^. This is especially puzzling when comparing microcline to other members of the feldspar group, which are both chemically and structurally closely related, but differ in their ice nucleation rates by orders of magnitudes^17–21^. These findings raise the question of which properties contribute to a material’s ability to promote ice nucleation.

To this end, several parameters have been discussed that are likely to be relevant when considering the ice-nucleating ability of a given material^22^. Historically, lattice match with a low-index face of hexagonal ice I_h_ has been brought forward as early as 1947, when Vonnegut presented his pioneering work on silver iodide^23^. Besides providing a template for nucleation and growth, the chemical affinity towards water—for example, through the presence of hydroxyl groups—has been argued to be important^24^. However, recent simulation studies modeling atomically flat, infinite surfaces have indicated that a single descriptor fails to reliably predict the ice nucleation ability on a microscopic level; instead, the combination of several features, such as the lattice symmetry and corrugation of the energy landscape, needs to be taken into account^25^. Experiments, on the other hand, have underscored the importance of mesoscale-sized active sites, such as step edges, cracks and pores^26–30^. These sites are known to be rare, as only a few of these sites are identified in large-scale microscope images^28^. We note that the term “active site” is used in two different ways in the literature. First, each position that nucleates ice is termed an active site. Within this definition, the number of active sites strongly increases with the ice saturation ratio^31^. Second, only rare and distinct positions, which nucleate ice at low saturation ratios, are termed active sites^28^. This notion implies that ice nucleation occurs preferentially at the same positions, i.e., the active sites, in subsequent experiments. In the following, we will use the second definition.

Kiselev et al. reproducibly observed 10–100 µm-sized ice clusters growing at mesoscopic step edges and cracks when cooling feldspar samples in a controlled humidity atmosphere^30^. These clusters exhibit a uniform orientation relative to the feldspar sample, providing compelling evidence for epitaxial growth. A lattice match between the secondary prism plane of ice I_h_ with the (100) surface plane of feldspar has been put forward as a possible explanation for the observed epitaxial growth^30^. The (100) surface is thermodynamically less stable than the (001) surface. However, surface features, such as cracks and step edges, might expose small facets of the (100) plane, providing an explanation as to why ice nucleation is preferred at these active sites on potassium-rich feldspar samples. Moreover, a comprehensive comparison of feldspar samples exhibiting different Na/K compositions as well as different microstructures suggests that the most active nucleation sites are similar across all alkali feldspars^32^. However, differences in the density and distribution of nucleation sites have been identified at low temperatures, where samples with a high surface ordering exhibit superior ice nucleation activity, again indicating that microcline outperforms the other feldspar types. As the size of the critical nucleus is of the order of nanometers^33–37^, a nanoscopic investigation is crucial to obtain an in-depth understanding of the nucleation process. So far, atomic-scale investigations showed that the most stable (001) surface of microcline is fully hydroxylated^38,39^. The hydroxyl arrangement can act as an adsorption position for water molecules^38,40,41^. Theoretical simulations addressing ice nucleation on microcline show contradictory results. Atomistic modeling suggests the possibility of stacking hexagonal ice with the primary prism face (10.0) on the (100) face of feldspar^30^, but direct molecular dynamics (MD) simulations do not observe ice nucleation on any low index plane on the accessible time scales^42,43^. Recent biased MD simulations, however, suggest that cubic ice could nucleate with the (110) plane stacked on the (110) surfaces of feldspar exposed at step edges^44^. Hence, a direct link between microcline’s exceptional ice-nucleating ability and its atomic surface structure remains elusive, hindering the development of an atomic-scale mechanistic understanding of ice nucleation on feldspar specifically and on mineral surfaces in general.

Here, we combine atomic-scale simulation results with mesoscopic freezing experiments by providing high-resolution images using dynamic atomic force microscopy (AFM) in ultrahigh vacuum (UHV). Microcline samples are cleaved in situ, cooled to 145 K and exposed to a water partial pressure in the order of 10^−8 ^mbar corresponding to a saturation ratio *S* ranging from 2 to 5. The growth of ice clusters is observed in situ on the nanoscale, enabling the direct identification of the nucleation sites. Repeated deposition and desorption experiments indicate that ice cluster formation is not limited to step edges but also occurs on the bare (001) terrace. Moreover, upon repeating the deposition cycles, the nucleation positions on the terrace scatter randomly, providing no evidence of preferential nucleation at active sites. We compare these results obtained on microcline with analogous experiments carried out on sanidine, a feldspar mineral with the same chemical composition as microcline, but with a different ordering of the aluminum atoms in the silicate framework. On sanidine, ice nucleation is observed primarily at the step edges, just as expected from the common notion of active sites. Both experimental findings are supported by MD simulations of hexagonal ice clusters, terminated by a (10.4) facet, on top of the microcline or sanidine (001) surface. Clusters appear to be more stable on microcline (001) than on sanidine due to the formation of several ice-like hydrogen bonds between the hexagonal ice lattice and hydroxyl groups of the microcline surface. Our work thus underscores the exceptional role of microcline as an ice nucleator at the nanometer scale by showing that its ice nucleation activity is not limited to active sites but also manifests on the bare terrace.

## Results

### Nanoscale observation of ice clusters on microcline (001)

**Fig. 1:**
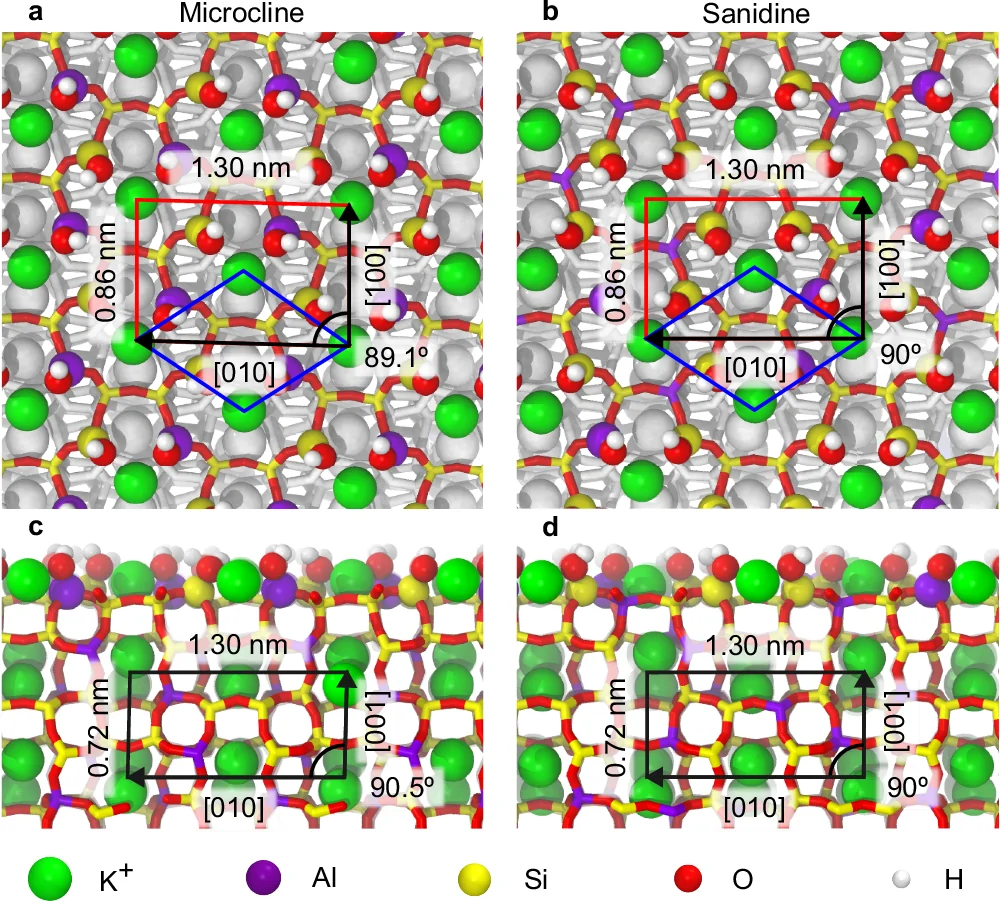
Crystal structure of hydroxylated microcline and sanidine according to MD simulations. **a**,**b** Top views on the (001) face. The centered (primitive) unit cell is shown in red (blue). **c**,**d** Side views along [001]. The aluminosilicate frameworks are shown as sticks with colors according to the legend. For clarity, bulk atoms are shown in gray in (**a**) and (**b**).

Feldspar minerals are built up by an aluminosilicate framework, which consists of (Al,Si)O_4_ tetrahedra bonded by shared oxygen atoms. Common counter ions are potassium, calcium and sodium. Here, we focus on potassium-rich feldspars with the sum formula KAlSi_3_O_4_. Microcline is the thermodynamically stable modification of potassium feldspar at room temperature, which is why it is also referred to as the low-temperature form. It has a triclinic crystal structure, in which the silicate framework has four distinct tetrahedral sites. In microcline, the aluminum atoms are located predominantly in one of these sites, resulting in an Al/Si-ordered structure^45^. The (001) surface geometry of microcline determined by MD simulations is shown in Fig. 1a, c. Each surface unit cell features two aluminol groups in crystallographically identical positions.

Sanidine is the high-temperature modification of potassium-rich feldspar, which is thermodynamically favored at the elevated temperatures of feldspar formation. Sanidine exhibits the same chemical composition and connectivity as microcline, but the aluminum atoms in sanidine are randomly distributed across all tetrahedral sites, resulting in an Al/Si-disordered structure. Due to the random distribution, the crystal symmetry increases compared to microcline, resulting in a monoclinic crystal structure^45^. In Fig. 1b, d the (001) surface geometry of sanidine is shown. While the unit cells of sanidine and microcline differ only by less than 1% in dimensions and less than 3% in the angle, the random distribution of aluminum atoms in sanidine results in only one, not two, randomly placed aluminol group per unit cell^45^, i.e., the density of surface aluminol groups is halved compared to microcline.

**Fig. 2:**
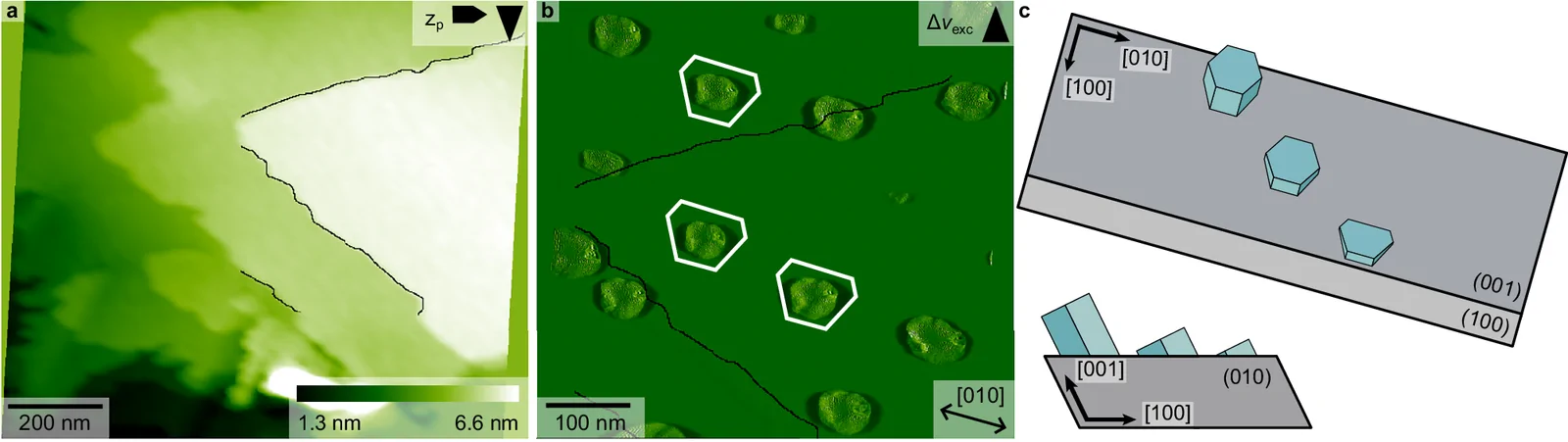
Nucleation of ice crystals on a freshly cleaved microcline (001) surface. **a** Topography image (z_p_) of the microcline (001) surface. Flat areas and step edges are seen. The arrows in the upper right corner mark the fast-scanning direction (trace) and slow-scanning direction (down). **b** Frequency shift image (Δ*ν*_exc_) of ice crystals grown on the upper right surface area in (**a**). The image was taken after increasing the water partial pressure to 6.8 ∙ 10^-8 ^mbar at 145 K corresponding to *S* = 4.5. Some crystals featuring an approximately hexagonal shape are marked by white distorted hexagons. The distorted hexagons result from the intersection of the hexagonal prism at an angle of 26 degrees, as observed by Kiselev et al^30^. Step edges which are imaged in (**a**) and (**b**) are marked by black lines. To reduce imaging artifacts on the cluster shape, an overlay of trace and retrace image is shown. In total, we performed 16 nucleation and desorption cycles at six different surface positions. The color scale in (**a**) applies to (**b**). **c** Schematic illustration of hexagonal ice crystals growing on top of the (001) with the orientation determined by Kiselev et al^30^. Due to the inclination angle and depending on the height of the crystal, the outer shape of the intersection varies as indicated by three exemplary crystals. The right crystal corresponds to the distorted hexagons shown in (**b**).

To investigate the ice nucleation on the thermodynamically most stable (001) face of microcline, we performed dynamic AFM measurements on samples cooled to 145 K in a UHV environment. At the nanoscale, the cleaved microcline (001) surface features several hundred-nanometer-wide terraces and a variety of different structures, such as steps and kinks. A representative view with triangular terraces is shown in Fig. 2a. In the lower part of the image, an area with many steps and cracks can be seen, while a comparatively flat area is seen in the upper right. For our experiments, we chose areas featuring both atomically flat regions and step edges. To induce ice nucleation, we dose water at pressures ranging from 2.9 to 7.2 ∙ 10^-8 ^mbar (corresponding to saturation ratios ranging from 2 to 5) using a leak valve while continuously scanning the surface. We are aware that the reported saturation ratios are significantly higher than the typically reported saturation ratios in macroscopic ice nucleation experiments. This difference is because we only image a small fraction of the surface. Consequently, we need higher saturation ratios to observe ice nucleation within the limited image frame on timescales of minutes. After ≈3-4 min, ice clusters of a few tens of nanometer height start growing on the surface at a minimal pressure of 4.6 ∙ 10^−8 ^mbar (see Supplementary Note 1, Supplementary Fig. 1). We note that initial clusters are observed both at step edges and on the bare terrace (see Supplementary Fig. 2). Then, the leak valve is closed, and we follow the shrinking of the ice clusters through desorption until they vanish. At this point, a new deposition and desorption cycle is performed, allowing comparison of nucleation sites at the same surface position. Images illustrating a full cycle are shown in the Supplementary Fig. 2. An AFM frequency shift image of ice clusters is presented in Fig. 2b. Unlike topography images, frequency shift images do not distinguish different heights but rather height changes. Hence, we have highlighted the position of the step edges, which are imaged in both images, by black lines as a guide to the eye. The measurement time for each image is 350 s.

Figure 2b shows two important characteristics of the grown ice clusters: First, the clusters do not exclusively nucleate at step edges but also grow on the bare terrace. Second, at least some of the clusters adopt an approximately hexagonal shape with similar orientation, which is a strong indication that the grown clusters are crystalline. Further, the orientation of these crystals perfectly matches that of the approximately 10–100 µm sized crystals observed by Kiselev et al. ^30^, as shown in the schematic illustration in Fig. 2c.

**Fig. 3:**
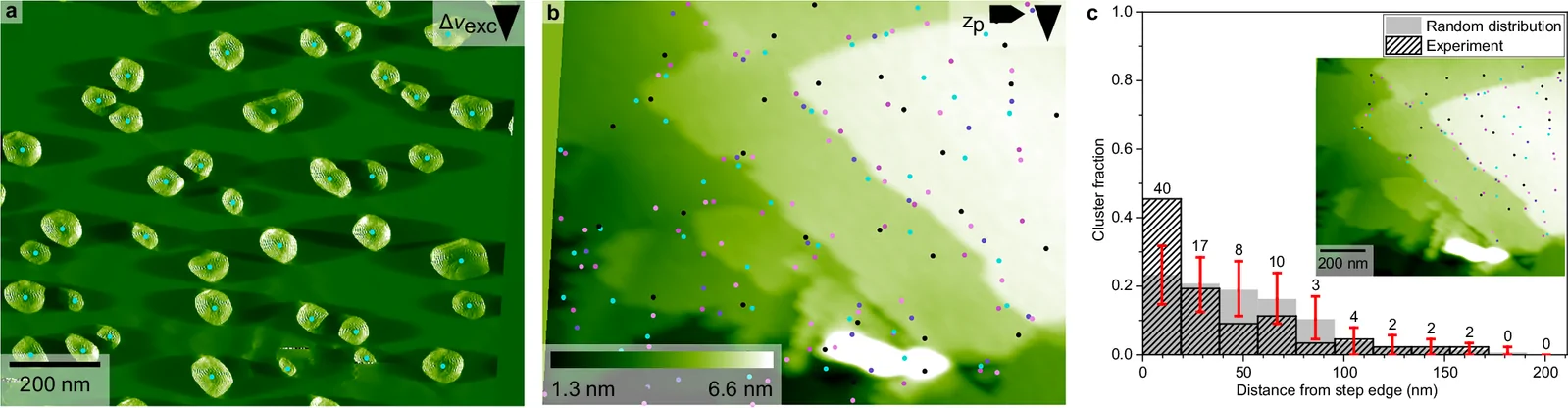
Sequential nucleation and desorption cycles on microcline. **a** Frequency shift AFM image of ice crystals grown in a single cycle. The image was taken after increasing the water partial pressure to 5.6 ∙ 10^-8 ^mbar at 145 K corresponding to a *S* = 3.7. Each individual cluster is marked by a centered light blue circle. The image is composed from a trace and a retrace image to reduce scanning artefacts. **b** Nucleation positions of five consecutive growth and desorption cycles superimposed on the topography image of the ice-free surface. Each color corresponds to one cycle. During the deposition period, the water partial pressure was raised to pressures between 5.3 and 7.2 ∙ 10^-8 ^mbar (*S* = 3.5-4.8). The scale bar in (**a**) applies to both images. **c** Distribution of the distance from the nearest step edge of the clusters marked in the inset. For comparison, a random distribution is shown by light gray bars. The error bars indicate the 95% credible interval of the random distribution (see Supplementary Note 3, Supplementary Fig. 6, 7). The inset AFM image shows all 88 analyzed nucleation positions from five consecutive nucleation and desorption experiments. The color scale in (**b**) also applies to the inset shown in (**c**). The number of nucleation events per bin is given above each bar. Source data are provided as a Source Data file.

To determine whether ice nucleation always occurs at the same, rare sites (which we then would classify as active sites) or is randomly distributed across the surface, we performed repeated cycles of nucleation and desorption at the same position (see Supplementary Note 2 and Supplementary Fig. 3, 4). For each cycle, we determined the center of each cluster as shown exemplarily in Fig. 3a. In Fig. 3b, the nucleation positions of five consecutive cycles are superimposed on a topography image of the bare substrate. These data show nucleation of ice clusters both at step edges and on the flat terrace, as noticed above. Moreover, the nucleation positions in different cycles are not at the same sites but are randomly distributed across the surface. The latter finding is important because it rules out the possibility that ice nucleation is induced by active sites on the bare terrace that were not identified in AFM images. We cannot rule out the presence of atomic defects, which could be responsible for the ice nucleation. In fact, many atomic defects are expected to exist on the surface. Nevertheless, if these defects were to act as nucleation sites, we would not classify them as active sites, as these defects are an intrinsic and frequent feature of the surface.

To quantify the preference of nucleation at step edges over the bare terrace, we determined the distance of clusters from the nearest step edge. The corresponding distance histogram in Fig. 3c reveals a distribution that differs only marginally from the distribution expected for random cluster nucleation. Directly at the step edge, the nucleation probability is higher than what is expected for a random distribution, indicating that the step edge does induce nucleation. However, we also observe a significant number of nucleation events at large distances from the step edge. This finding demonstrates that ice nucleation also occurs on the bare terrace. To confirm whether this near-random distribution of nucleation positions also holds for individual cycles, we also show the histograms of each individual cycle in Supplementary Fig. 5. As the saturation ratio in each cycle differs, comparing these histograms reveals information regarding the saturation ratio dependence of the observed ice nucleation: while we observe a preference for the nucleation at step edges at the lower observed saturation ratios, the distance distribution at the highest saturation ratio perfectly matches a random distribution. Nevertheless, across all investigated saturation ratios, we observed ice nucleation at step edges and on the bare terrace.

### Comparison with sanidine (001)

**Fig. 4:**
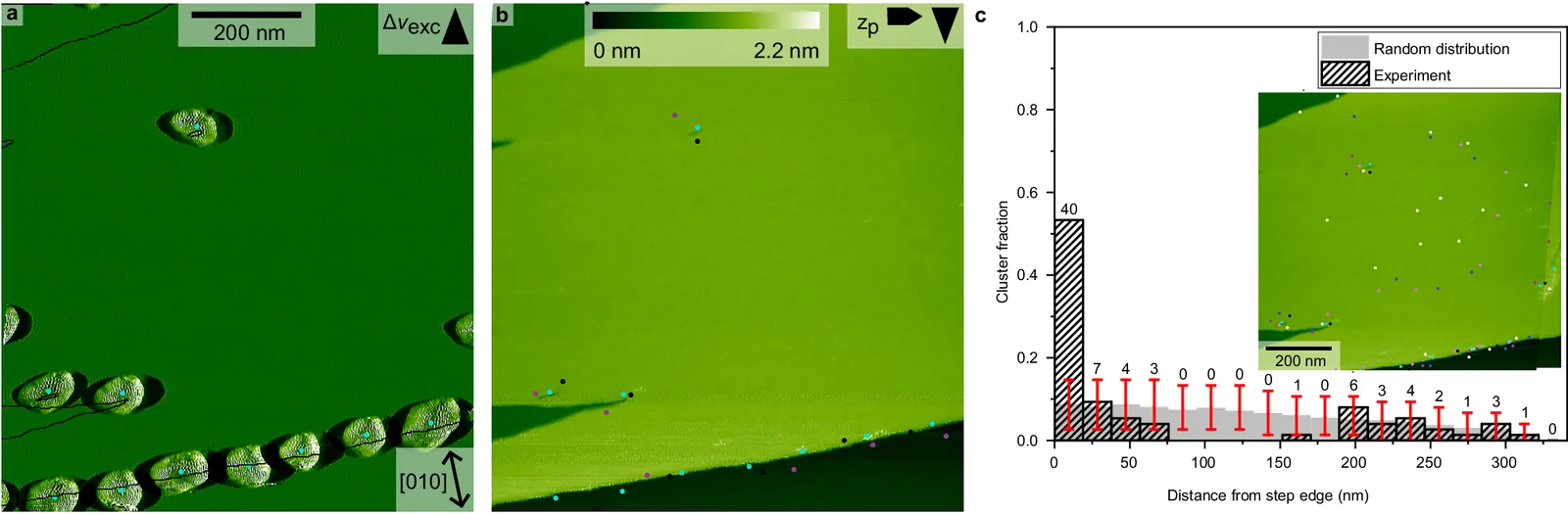
Nucleation of ice crystals on the sanidine (001) surface. **a** Frequency shift AFM image of ice clusters grown in one cycle on sanidine at pressures up to 8.4 ∙ 10^−7 ^mbar (S = 5.6). **b** The positions of ice nucleation of three consecutive cycles of nucleation and desorption superimposed on the topography image of the ice-free surface. Each color corresponds to one cycle. The scale bar in (**a**) applies to both images. **c** Distance from the nearest step edge distribution of the clusters marked in the inset. We also show a distribution that would occur if the clusters were randomly distributed (light gray bars) for comparison. The error bars mark the 95% credible interval of the random distribution. The inset AFM image shows all 75 analyzed nucleation positions from six consecutive nucleation and desorption experiments. Nucleation on the bare terrace is only observed in cycles with higher water partial pressures (see Supplementary Fig. 8). The color scale in (**b**) also applies to the inset shown in (**c**). The number of nucleation events per bin is given above each bar. Source data are provided as a Source Data file.

To determine whether these findings are specific to microcline, we performed analogous experiments on sanidine (001) at pressures ranging from 5.6 ∙ 10^-8 ^mbar to 1 ∙ 10^-7 ^mbar. At pressures above 7 ∙ 10^-8 ^mbar, we observed ice nucleation. These experiments shown in Fig. 4a, b reveal that nucleation almost exclusively occurs at step edges for water pressures below 9 ∙ 10^-8 ^mbar. Only at higher pressures, clusters start to form on the bare terrace of sanidine (see Supplementary Fig. 8).

The preference for nucleation at step edges on sanidine is also revealed in the step edge-cluster distance histogram in Fig. 4c, which strongly deviates from a random distribution. Ice clusters are five times more likely to nucleate directly at a step edge than expected based on a random distribution. Interestingly, the experimental data reveal a bimodal distribution. This is most likely because a growing ice cluster reduces the water vapor density in its surroundings, thereby reducing the probability of further nucleation events in its direct neighborhood. Hence, ice clusters do either nucleate close to the step edges or at larger distances, resulting in the observed bimodal distribution. Comparing the histograms of individual cycles shown in Supplementary Fig. 9 underlines that ice nucleation exclusively occurs at step edges at the lowest observed supersaturation ratios (which are still high compared to the supersaturation ratios at which ice nucleates on microcline). Only at even higher supersaturations is a bimodal distribution observed.

Note that the water partial pressure required to induce ice nucleation on microcline and sanidine depends on the sample temperature, as the vapor pressure of ice is a function of temperature (at 145 K, the hexagonal ice vapor pressure is 1.51 ∙ 10^-8^ mbar^46^). To account for this, we performed all experiments presented here with the sample maintained at a constant temperature of 145 K. Further, as the water partial pressure is gradually increased, ice nucleation may be observed on any surface maintained at 145 K. A sample with high ice-nucleating ability, however, requires substantially lower pressure than a sample with poor ice-nucleating ability. In the usual model, where defects and step edges are believed to facilitate ice nucleation, ice clusters form at step edges at considerably lower pressures than on defect-free terraces. This is exactly what we observe on sanidine. At a pressure of about 7 ∙ 10^-8 ^mbar, the first ice clusters are seen to grow preferentially at step edges.

On microcline, however, the picture is markedly different. First, the minimal pressure of about 4.6 ∙ 10^-8 ^mbar required to observe ice nucleation on microcline is lower than the minimal pressure required for sanidine. This already demonstrates the superior ice-nucleation ability of microcline compared with sanidine. Second, at the minimal pressure on microcline, ice clusters already nucleate at both the step edges and on the bare terrace.

These observations indicate that the atomically flat surface of microcline (001) is active in ice nucleation, even in the absence of specific active sites. The latter finding is in contrast to the prevailing view of special sites on microcline being responsible for the high ice nucleation activity of microcline^28–30^. We note that ice nucleation on microcline can, of course, also occur at active sites. The essential point here is that even the atomically flat (001) face of microcline exhibits superior ice nucleation ability at the saturation ratios investigated here. We are aware that atmospheric conditions differ from those in our low-temperature UHV setup. However, given the fact that the (001) surface is the thermodynamically most stable face of microcline, our findings might be relevant for understanding the exceptional role of microcline as ice-nucleating particles at higher temperatures and lower saturation ratios in the atmosphere at the nanoscale.

### Molecular-scale simulations

To rationalize the experimental observation that the ice crystals do not grow with their basal plane parallel to the (001) face, we compared different unit cells of the (001) face of microcline with projections of higher index planes of ice I_h_. Based on this comparison we identified the best lattice match of better than 5% between a microcline unit cell spanned by the vectors [310] and [010] and the (10.4) surface of ice I_h_ (see also Supplementary Note 4, Supplementary Tables 1-3). This unit cell match is a first hypothesis, but it is not sufficient to explain the difference in ice nucleation on microcline and sanidine, as their unit cell dimensions differ only slightly (cf. Fig. 1). The major difference between the (001) faces of microcline and sanidine is the density and ordering of aluminol groups. While microcline (001) is terminated by two aluminol groups per unit cell, arranged in a periodic pattern, sanidine has, on average, only one randomly-placed aluminol group per unit cell. Since aluminol groups bind water stronger than silanol groups^41^, the higher density and periodic placement of aluminol groups on microcline might facilitate ice nucleation.

**Fig. 5:**
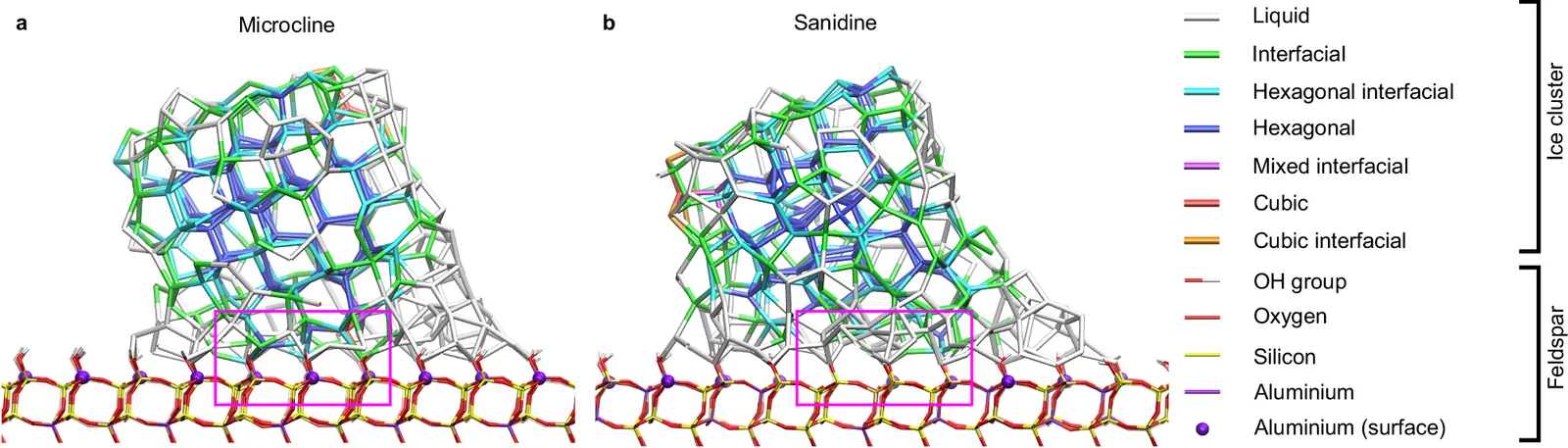
MD simulations of hexagonal clusters on microcline and sanidine (001). Snapshots of MD simulations at 145 K of hexagonal ice clusters, cut along the (10.4) plane and placed at similar positions on microcline (**a**) and sanidine (**b**). The network of water oxygen atoms within the ice clusters and surface hydroxyl oxygen atoms is color-coded according to the local structure determined by the LICH-TEST algorithm. The cluster placed on microcline (001) forms ice-like hydrogen bonds to several surface hydroxyl groups, while the cluster on sanidine (001) barely forms ice-like hydrogen bonds to surface hydroxyl groups (magenta rectangles). Further details are provided in Supplementary Note 5 and Supplementary Figs. 10, 11.

To test this hypothesis, we performed MD simulations at *T* = 145 K of a proton-disordered nanoscale ice I_h_ crystal^47^, terminated by a (10.4) facet, and placed on microcline (001) or sanidine (001). On both feldspar surfaces, we chose two different cluster positions, determined by the symmetry of the hydroxyl groups. We monitored the number of ice-like water molecules in the system with the LICH-TEST^48^ until an equilibrium was reached. On microcline, the fraction of ice-like water molecules was higher than on sanidine, indicating a higher stability. Further, the hexagonal ice network of the cluster placed on microcline (001) forms ice-like hydrogen bonds to several hydroxyl oxygens on the microcline surface, as shown in Fig. 5. This finding suggests that these hydroxyl groups could act as a template during hexagonal ice nucleation. Thus, our simulations support the proposed mechanism of stacking ice I_h_ with its (10.4) surface on feldspar (001), which explains the orientation of the ice crystals in the experimental AFM images in Fig. 2. While these simulations do not reveal the atomistic ice nucleation mechanism directly, the differences in ice binding observed are in agreement with the experimental evidence presented above that ice nucleation on microcline (001) occurs more readily than on sanidine (001).

## Discussion

In this work, we aimed to obtain nanoscale mechanistic insights into ice nucleation at feldspar mineral surfaces in order to shed light on the exceptional ice nucleation ability of microcline. Nanoscale imaging inherently implies that only a small fraction of the surface can be imaged. Therefore, higher saturation ratios are needed as compared to, e.g., light microscopy studies that cover a macroscopically large surface area. Thus, the conditions used in the present experiments are inherently different from those typically found in the atmosphere. Accordingly, the obtained results should not be interpreted as a direct representation of atmospheric ice nucleation. Rather, they should be viewed as a mechanistic framework that complements studies conducted under more atmospherically relevant conditions. Such studies have consistently demonstrated that ice nucleation is dominated by active sites, which can be associated with mesoscale topographic features, such as cracks and pores^27,28,30^. However, while these approaches identify the importance of topographic features, they are not able to provide insights into the underlying, nanoscopic processes that govern ice nucleation at the molecular level. In contrast, theoretical studies typically address ice nucleation at the molecular scale, but often lack direct experimental verification^22,24,25^. In this context, the present study provides a missing link between mesoscale experimental observations and molecular-level theoretical modeling. Because high-resolution imaging within surface features, such as cracks and pores, is currently not feasible, we are restricted to less active surface regions, such as flat terraces and small step edges. Nevertheless, mesoscale surface features consist of distinct facets at the nanoscale. In particular, because the (001) surface of microcline is the most stable one, it is plausible that this surface is also exposed within crack and pore structures. Our results unambiguously demonstrate that even the bare (001) surfaces of microcline and sanidine, despite being structurally very similar, exhibit significantly different ice nucleation efficiencies. On microcline (001), ice nucleation is observed already at a minimal pressure of 4.6 ∙ 10^-8 ^mbar and occurs almost randomly scattered across the surface with only a small preference for step edges at the investigated conditions. In contrast, on sanidine (001), a substantially higher pressure is necessary to induce ice nucleation, which occurs predominantly at step edges and only marginally on the bare terrace.

We further note that on both microcline and sanidine, the preference for nucleation at step edges increases with decreasing saturation ratio. This finding is fully consistent with previous optical microscopy experiments showing that, at low saturation ratios, ice nucleation is restricted to a small number of active sites, whereas at higher saturation ratios it occurs at a larger number of more randomly scattered positions^29^.

Our findings do have important implications: First, they show that even minor atomic-scale variations can translate into substantial differences in the ice nucleation efficacy. Thus, the different ice nucleating behavior of two closely related minerals, such as microcline and sanidine, may be induced by only subtle structural differences. For microcline and sanidine, this difference in ice nucleation behavior can most likely be attributed to the higher aluminol density in microcline. Second, as discussed above, active sites, such as pores, are composed of facets, such as the (001) surface investigated here. It has previously been demonstrated that ice nucleation in pores is strongly enhanced by the inverse Kelvin effect, which promotes water condensation even below water saturation, followed by freezing^49^. If the atomic scale structure were irrelevant under such conditions, the ice crystals emerging from these active sites would not be expected to exhibit any preferred orientation. This is, however, in contrast to experiments on the mesoscale, clearly showing well-oriented ice crystals growing repeatedly at the same active site^30^. Taken together, these observations indicate that atmospheric ice nucleation cannot be understood in terms of mesoscale topography or molecular-scale structure alone. Rather, a combination of both effects has to be considered, as the topographic enhancement associated with the inverse Kelvin effect and the molecular-level influence of crystallographic match are likely to act cooperatively.

Moreover, the observation that the ice crystals grown in our experiment exhibit a similar orientation and shape to those reported for crystals nucleated at active sites in the study by Kiselev et al. suggests the conclusion that (001) faces within cracks and pores are responsible for the observed nucleation behavior. This interpretation appears perhaps more compelling than attributing the high ice nucleation efficacy to the high-energy (100) face, which is thermodynamically less stable and therefore expected to occur only in negligible amounts, if at all.

The performed molecular-scale simulations indicate that hexagonal ice clusters, terminated by a (10.4) facet, are more stable on microcline (001) than on sanidine (001). In particular, the hexagonal ice network forms ice-like hydrogen bonds to several hydroxyl oxygens on the microcline surface, suggesting that these hydroxyl groups could act as a template during ice nucleation.

To conclude, our findings underscore the exceptional role of microcline in ice nucleation, as they provide evidence that microcline’s higher ice nucleation efficacy than sanidine already manifests in the absence of active sites on the bare, most stable (001) surface. Hence, the exceptional ice-nucleating efficacy of microcline can be attributed at least in part to its atomic structure and is not exclusively determined by mesoscopic topographic properties. Further, the growth of crystals, which are oriented with a higher index plane parallel to the (001) surface, emphasizes that crystallographic match, which enhances the ice nucleation efficacy, is not limited to the commonly investigated low-index planes of ice, which, in our opinion, is of general importance.

## Methods

### Materials

Cuboidal samples cut from microcline and sanidine feldspar were obtained from SurfaceNet (Germany). The samples have a base size of 5 × 5 mm and a height of 5–6 mm before cleavage. The base plane is oriented parallel to the (001) crystal plane, allowing the samples to be cleaved along one of the base sides to expose a fresh (001) crystal surface.

Microcline samples from the same crystal have been characterized previously by X-ray diffraction (XRD)^39^, chemical analysis^40^, as well as optical and electron microscopy^50^. Chemical analysis has revealed that the microcline samples consist of approximately 96% potassium feldspar, 4% sodium feldspar and less than 0.1% calcium feldspar. Trace element mass concentrations are Ba (32 ppm), Sr ( < 5 ppm), Rb (12 ppm), Cu (119 ppm), Fe (0.10 wt.%) and Pb ( < 5 ppm). With XRD, it was confirmed that the samples exhibit a high degree of Al/Si order with 80% of aluminum atoms situated in T1(o) sites. The presence of a perthitic structure (exsolution into potassium- and sodium-rich domains) was confirmed by scanning electron microscopy with energy-dispersive X-ray spectroscopy. Further information on the sample characterization can be found in the respective publications.

The sanidine sample originates from Madagascar. The sanidine single crystal of AlK_0.85_Na_0.14_O_8_Si_3_ was examined on a Rigaku Supernova diffractometer using Mo *K*_α_ (*λ* = 0.71073 Å) radiation. The crystal was kept at 100.0(1) K during data collection. Using Olex2^51^, the structure was solved with the SHELXT^52^ structure solution program using Intrinsic Phasing and refined with the SHELXL^53^ refinement package using Least Squares minimization. The K: Na ratio was refined to 85:15. The ratio of Al: Si cannot be refined reasonably; it was fixed to 1:1 for Si2A:Al1B. K1A/Na1B and Si2A/Al1B were constrained to have equivalent thermal motion.

Based on XRD data reported in Supplementary Note 6, lattice parameters in the space group C 2/m are *a* = 8.5586 Å, *b* = 13.0070 Å, *c* = 7.2037 Å, *β* = 116.052°. The sample consists of around 85% potassium feldspar and 15% sodium feldspar. The amount of calcium feldspar was not determined. Parametric analysis of the lattice parameters as described by Kroll and Ribbe^54,55^ reveals a total T1 site fraction for aluminum of ΣT1 = 0.731, which corresponds to low sanidine according to the classification of Ribbe^56^.

The sanidine surface, which was cleaved in ultrahigh vacuum (UHV), was investigated with an optical microscope (DCM8, Leica, Germany), see Supplementary Note 7. An overview image of the entire surface was produced by stitching together 20 images at 10x magnification. Based on this image, details of the surface were selected and imaged with 50x and 150x magnification.

### AFM measurements

We performed all AFM experiments under UHV conditions with a base pressure well below 10^-10 ^mbar. The feldspar samples (SurfaceNet) were transferred into the UHV system after degassing the samples at 400 K overnight in a load lock. After further degassing at 600 K for at least 1 h in UHV the crystals were cleaved in situ. If necessary, surface charges were reduced by annealing at 450 K overnight. Afterward, the samples were transferred into the AFM (Omicron AFM VT XA; Omicron, Germany). The AFM was cooled with liquid nitrogen to achieve a temperature of 145 K, measured on the tantalum sample holder as close to the crystal surface as possible. We expect that the surface temperature of the samples is equal to or higher than 145 K. To minimize the temperature deviations between samples, we cleaved all samples at the same height. To control the water pressure inside the UHV system, we use a leak valve oriented directly onto the crystal surface inside the AFM. The water (stakpure) was purified with multiple freeze-pump-thaw cycles. We confirmed the purity with mass spectrometry.

We want to emphasize that we had to optimize between scan parameters suitable for imaging several hundred picometer high steps and parameters to image several tens of nanometer high ice clusters. This results in an elongated cluster shape in the fast-scanning direction as the feedback loop is too slow to approach the flat surface at the end of the cluster. However, the real cluster shape can be revealed by comparing images of both fast-scanning directions (trace and retrace). To this end, we adopted the same strategy as presented previously by Ebeling et al. ^57^ and added the trace and retrace image. All AFM images are drift-corrected with unDrift using stationary features^58^. Further, we performed a plane subtraction (three-point level) on topography images using Gwyddion^59^. To obtain the cluster positions, we manually determined the outline of each cluster and detected the corresponding centroid with Fiji^60^ (see Supplementary Note 3 and Supplementary Fig. 6). To determine the expected random distribution, we manually identified the clearly visible step edges. Next, we created a distance map using the distance transform based on chamfer distances of the MorphoLibJ library^61^ for Fiji. The gray value distribution of the obtained image corresponds to a random distribution of nucleation positions (see Supplementary Note 3 and Supplementary Fig. 6).

### MD simulations

We performed atomistic MD simulations of hexagonal ice clusters on microcline and sanidine using the LAMMPS code^62^. Feldspar-water interactions were described with the CLAYFF forcefield^63^, using modified charges for feldspar from Kerisit et al. ^64^ and including the optional three-body potentials for silanol (SiOH) and aluminol (AlOH) groups on the surface. While the original CLAYFF forcefield employed the SPC water model, here we used the TIP4P/Ice water model^65^ instead, as it produces the correct melting point and coexistence curve between liquid water and hexagonal ice, unlike the SPC model. Systems were simulated in 3D periodic boundary conditions. Lennard-Jones interactions and real-space electrostatic interactions were cut off at 0.85 nm, long-range electrostatics were calculated using the particle-particle particle-mesh solver (pppm/tip4p). The unit cell parameters for microcline and sanidine were taken from Bailey^66^, and Angel et al.^67^, respectively. After an initial energy minimization, we used a velocity Verlet integrator with a time step of 1 fs to integrate the equations of motion for 10 ns, and a Nosé-Hoover chain thermostat of length 3 with a time constant of 0.1 ps was used to simulate a canonical ensemble (*NVT*) at a temperature of *T* = 145 K. Atomic positions were stored every picosecond for subsequent analysis with the GPTA code^68^ and the LICH-TEST algorithm^48^. Visualizations were done in VMD^69^. Further details are provided in Supplementary Note 5.

### Reporting summary

Further information on research design is available in the Nature Portfolio Reporting Summary linked to this article.

## Supplementary information

Supplementary Information

Supplementary Data

Reporting Summary

Transparent Peer Review file

## Source data

Source Data

## Data Availability

The data that supports the findings of this study are available from Figshare^70^ and from the corresponding authors upon request. Crystallographic data are available from the joint Cambridge Crystallographic Data Center (CCDC) and Fachinformationszentrum Karlsruhe Access Structures service under deposition number 2532369. Unprocessed raw data, including AFM and MD data, are provided as Supplementary Data 1. Source data are provided with this paper.

## Code availability

Open-source software was used to analyze the AFM data^58–61^. XRD data was analyzed with free for academic use software^51–53^. Software used for the MD simulations is also open source^62,69^.
