# Supplementary material for: Ice nucleation on microcline (001) in the absence of active sites
Source: Nat Commun. 2026 Aug 25;17:8652. doi: 10.1038/s41467-026-76548-7 (PMC13507223; doi:10.1038/s41467-026-76548-7)
Supplement: Supplementary file 1 [file 41467_2026_76548_MOESM1_ESM.pdf]

Supplementary Information

Ice nucleation on microcline (001) in the absence of active sites

Florian Schneider, Rasmus Väinö Erik Nilsson, Ralf Bechstein, Hans-Georg Stammler, Bernhard Reischl, Thomas Koop, Angelika Kühnle and Tobias Dickbreder

Email: fschneider8@uni-bielefeld.de; tobias.dickbreder@univie.ac.at

This PDF file includes:

Supplementary Notes 1-7

Supplementary Figs. 1-12

Supplementary Tables 1-3

Supplementary References

Supplementary Note 1: Further details on nucleation cycles

To observe the nucleation of ice clusters and determine the nucleation positions, we performed several nucleation cycles at the same positions.

Each cycle consists of the following steps:

1. We opened the leak valve and imaged the growth of ice clusters.
2. After a few minutes the ice clusters arrived at heights of a few tens of nanometers, and we closed the leak valve.
3. The ice crystals shrank until the free surface remained and the next cycle can be done.

The pressure development of one exemplary dosing cycle is presented in Supplementary Fig. 1. After opening the leak valve, the water pressure sharply rises followed by a continuous increase. After closing the leak valve, the pressure decays exponentially.

The measurement procedure of one cycle is illustrated in Supplementary Fig. 2. After choosing a suitable surface area, we retract the tip (black area in Supplementary Fig. 2a) to open the leak valve to the desired water pressure (in this particular case of $4.7 \cdot 10^{-8}$ mbar). While we image the nucleation and growth of ice clusters the pressure gradually rises (in this particular case up to $5.6 \cdot 10^{-8}$ mbar). To close the leak valve, we retract the tip again (black area in Supplementary Fig. 2b). Afterwards, we image the grown clusters to determine their positions. The vapor pressure decreases below the vapor pressure of ice, resulting in desorption of these clusters. In the upper part of Supplementary Fig. 2c all clusters are already desorbed. Please note that each image takes 6 minutes and the slow scanning direction is marked by a black arrow pointing up or down.

Supplementary Note 2: Further experimental AFM data of nucleation cycles

We performed ice nucleation experiments on two microcline and one sanidine crystal. Each crystal was cleaved only once as thin as possible within our sample holder to minimize the deviation between surface temperature and the temperature measured by the thermocouple attached to the sample holder.

In total, we performed 16 nucleation and desorption cycles at six different sample sites on microcline. In each cycle we observed that ice nucleation is not limited to step edges but occurs also on the bare surface.

On sanidine we performed 13 nucleation and desorption cycles at two different sample positions. (In another experiment we performed another eight cycles at one sample position. However, the sample temperature was about 3 K higher than in the other experiment, resulting in a higher pressure ($p > 1 \cdot 10^{-7}\,\mathrm{mbar}$) necessary to induce ice nucleation. This is why this series was neglected for this analysis.) In all these experiments, we observed ice nucleation predominantly at step edges. Ice nucleation at the bare terrace was exclusively observed for the highest supersaturations. We want to note that the imaged shape of the crystals is sensitive to the quality of the used AFM tip, often resulting in a broad and blurred mapping of the grown ice crystals. Further, the simultaneous imaging of both the ice crystals and surface features such as step edges, is experimentally challenging and requires a sharp tip. Nevertheless, this simultaneous imaging is necessary to determine the distance between the center of

each ice crystal and the closest step edge. Moreover, to compare the difference in nucleation between bare terrace areas and step edges, we need AFM images showing also large terrace areas. For images showing only areas with small terrace widths, the corresponding distance distribution would be limited to only small distances. Hence, we carefully chose only those cycles that provide simultaneous imaging of both well-defined ice crystals and large terrace areas, which were five out 16 for microcline and six out of 13 for sanidine.

The corresponding AFM images of ice clusters grown on microcline (sanidine) are shown in Supplementary Fig. 3, 4 (Fig. 8). Each figure shows the trace and retrace images along with the added image, in which the real cluster shape is revealed and a topography image on which the center of each cluster is marked.

Supplementary Note 3: Distance histograms of the distance between step edges and nucleation positions

In the following, we describe the routine to obtain the distance histogram shown in the main text, which reveals the distances between step edges and the nucleation positions. We manually determined the outline of each cluster and detected the corresponding centroid as shown in Supplementary Fig. 6a with Fiji.[1]

Furthermore, we measured the distance between each centroid and the nearest step edge (see Supplementary Fig. 6b). For the distance histogram, we only considered centroids whose distance to the nearest step edge could be determined unambiguously. Therefore, we excluded areas that were closer to the image border than to the nearest step edge as well as areas in which no unambiguous determination of the nearest step edge was possible (lower part of Supplementary Fig. 6b). In addition, we exclusively analyzed the area imaged by all consecutive measurement cycles. This results in the area marked by a light blue outline in Supplementary Fig. 6b.

To determine the expected random distribution, we manually identified the clearly visible step edges as presented in Supplementary Fig. 6c. Additionally, we created a distance map using the distance transform based on chamfer distances of the MorphoLibJ library for Fiji .[2]

The latter tool determines the approximate Euclidean distance of each pixel from the marked step edge. The grey value of each pixel in the generated image (Supplementary Fig. 6d) is proportional to the distance from a step edge. Brighter areas are farther away from the step edges. Accordingly, the grey value distribution corresponds to a random distribution of nucleation positions.

The probability that *n* clusters nucleate in an area in a specific distance interval *i* from step edges is given by

$$P_n^i = {p_i}^n (1 - p_i)^{N-n} \binom{N}{n} \tag{1}$$

with N being the total cluster count and

$$p_i = \frac{a_i}{A} \tag{2}$$

with the area of the chosen distance interval a_i and the total area A. The probability distributions of each distance interval are given in Supplementary Fig. 7a. The height of each bar corresponds to the expectation value. In Supplementary Fig. 7b the 95%

credible interval of the first probability distribution is given. The 95% credible interval was marked in the distance distribution as error bars (Supplementary Fig. 7c).

The obtained step-cluster distance distributions obtained for each individual cycle on microcline (sanidine) are presented in Supplementary Fig. 5 (9). In total, we evaluated 88 (75) nucleation events on microcline (sanidine). The comparison of the distributions obtained for different saturation ratios reveals that at most investigated saturation ratios the nucleation rate directly at the step edges is higher than expected for a random distribution by a factor of about two. However, we also observe ice nucleation on the bare terrace. At the largest observed supersaturation, the experimentally observed distribution does not deviate from a random distribution.

In contrast, on sanidine, the experimentally observed distribution strongly deviates from a random distribution at all investigated saturation ratios. At lower saturation ratios, which are higher than the saturation ratios on microcline, we observe ice nucleation exclusively at step edges. At higher saturation ratios, we observe a bimodal distribution: ice nucleation is predominantly observed directly at the step edges and, to a lesser degree, at distances larger than 100 nm from the step edges, but never in between. This bimodal distribution indicates that the nucleation occurs predominantly at step edges resulting in a lower water concentration in the vicinity of the step edges. As a consequence, the decreased water concentration reduces the probability of further nucleation events close to the step edges. These findings indicate that the nucleation rate on sanidine on the bare terrace is much smaller than the nucleation rate at steps.

Supplementary Note 4: Search for lattice match between microcline (001) and ice I_h

On microcline (001), we observe the formation of ice clusters on the bare, featureless terrace. The absence of features (especially steps and cracks) on the terrace suggests that the clusters in our experiment nucleate directly on the (001) surface of microcline and not on some other facets exposed in cracks or pores. To rationalize this observation and identify candidate structures for subsequent MD calculations, we searched for a potential lattice match between microcline (001) and hexagonal ice I_h.

As discussed in the main text, the shape and orientation of the clusters agree with the shape previously observed by Kiselev et al.[3] for macroscopic ice crystals growing on potassium feldspar (001) and (010) surfaces in deposition mode. Kiselev et al. explained this orientation with epitaxy between the {10.0} plane of ice and the (100) plane of potassium feldspar as exposed in cracks or pores. While this model cannot explain the nucleation on the (001) surface in our experiments, we used the proposed epitaxy between feldspar (100) and ice {10-10} as a starting point for our structure search.

The (100) and (001) surfaces of feldspar share the [010] direction (b_f = 1.30 nm), which has a good lattice match with the <11-20> direction of ice (a_{ice} = 0.45 nm, [4]), where $b_{\mathrm{f}}/a_{\mathrm{ice}} = 2.88$. Alignment between the feldspar [010] and ice <11.0> directions is also critical to achieve the orientation of the ice crystals observed in the AFM images. Based on both arguments, we assume that the ice <11-20> direction lies within a potential matching lattice plane, which leaves us with the set of {*h* 0 -*h* l} lattice planes as candidate planes.

To further narrow down the list of candidate planes, we compare the dimensions of the parallelogram created by intersecting the ice unit cell with the (*h* 0 -*h* l) lattice plane to the angles and lengths of the [*h’ k* 0] directions on the feldspar (001) surface. We

focused especially on the similarity of angles, because differences in angles cannot easily be compensated for by increasing the size of the unit cell. The properties of the low-index planes and directions considered for this analysis are given in Supplementary Tables 1 and 2.

Based in the information given in Supplementary Tables 1 and 2, we identified that the angle enclosed by the microcline [010] and [310] directions is close to the parallelogram angles of the (10-14), (10-15) and (10-16) planes of ice (highlighted in red in Supplementary Tables 1 and 2). Further, the angle between the [010] and [410] directions of microcline and the parallelogram angle of ice (10-12) is similar (highlighted in blue in Supplementary 1 and 2). To further narrow down the list of candidate structures, we compared the dimensions of the structures (see Supplementary Table 3), which shows the best match for the ice (10-14) plane with a microcline cell defined by the [010] and [310] directions. In this case, the difference in angle is only 1.55%, and the ratios of the cell dimensions very close to integer values (3.01 and 2.88, respectively).

Supplementary Note 5: Further details on the MD simulations

To analyze the stability of hexagonal ice clusters on microcline and sanidine surfaces, we constructed surface slabs measuring 6×4 conventional unit cells along the [100] (*x*) and [010] (*y*) directions, respectively, exposing the (001) surface along the *z* direction of the simulation box. The thickness of the slabs was three unit cells and both surfaces of the slab were α-terminated and fully hydroxylated. The perfectly ordered maximum microcline system was generated by replicating the unit cell. For the disordered sanidine system, we wrote a pseudo-Monte Carlo code to generate multiple slabs with random distributions of the Al substitutions satisfying Löwenstein's rule[5] of avoidance (no Al substitutions in corner-sharing (Al,Si)O_4 tetrahedra) and chose the slab with the smallest differences between the number of Al substitutions per layer, to avoid large surface dipole moments.

The ice clusters were generated from a proton-disorder hexagonal ice structure using the GenIce code,[6] and cut to expose basal and primary prism faces. The original ice cluster, containing 432 water molecules and measuring about 3.0 nm × 2.5 nm × 2.5 nm, was then cut at the bottom along the (10.4) plane, and rotated to align the (10.4) plane of ice I_h parallel to the (001) plane of feldspar, and the [21.0] direction of ice I_h parallel to the [010] direction of feldspar, as suggested by the lattice match in Supplementary Table 3. The ice clusters were then shifted to two different positions on the feldspar (001) surfaces, matching the position of an oxygen atom in the ice lattice to that of a surface hydroxyl group oxygen atom, and water molecules in the truncated ice cluster overlapping with the surface were removed. The final size of the ice clusters was 338 and 348 water molecules for positions 1 and 2, respectively. Simulation boxes measured about 5.2 nm × 5.2 nm × 6.7 nm, leaving more than 2 nm vacuum gaps between periodic images of the ice clusters along x and y directions, and between the top of the ice cluster and the periodic image of the bottom of the feldspar slab along the z direction.

The local structure of water molecules in the ice clusters during the MD simulation was analyzed with the LICH-TEST algorithm.[7] We included the oxygen atoms of surface hydroxyl groups in the analysis, to check for connections between the ice I_h lattice and hydroxyl groups. For all systems, the count of water molecules in ice-like configurations initially drops, as the interface between cluster and surface equilibrates and water molecules at the free surfaces of the cluster form the expected pre-melted layer. Supplementary Fig. 10 shows representative snapshots of ice clusters after 8-9 ns

equilibration. The last frames of each trajectory are provided as PDB files as part of the Supplementary Data. The cluster in position 1 on microcline remained in that position but the c-axis became tilted, due to pre-melting at the interface. Sometimes direct connections between ice and surface hydroxyls are registered. On sanidine, the ice cluster moves away from position 1, there is more disorder at the interface compared to microcline, and no direct connections between ice and surface hydroxyls are registered. The cluster in position 2 on microcline remains in its position and less disorder at the interface is observed compared to position 1, with several persistent direct connections between the ice lattice and surface hydroxyl groups. On sanidine, the ice cluster moves away from position 2, there is some pre-melting at the interface. Sometimes direct connections between ice and surface hydroxyls can be observed, but they are less persistent than on microcline. Supplementary Fig. 11 shows the average number of surface silanol and aluminol oxygens identified as part of the ice network, highlighting the good stability of the ice cluster at position 2 on microcline (001), which suggests a possible atomic-scale mechanism for ice nucleation on this surface, where certain hydroxyl groups can act as anchors for hexagonal ice.

Supplementary Note 6: X-ray diffraction measurements

Crystal Data for $AlK_{0.855}Na_{0.145}O_8Si_3$ (M =276.01 g mol^{-1}): monoclinic, space group I2/m (no. 12), a = 7.2037(3) Å, b = 13.0069(5) Å, c = 8.4255(4) Å, β = 114.134(6)°, V = 720.45(6) Å^3, Z = 4, T = 100.0(1) K, μ(Mo Kα) = 1.296 mm^{-1}, D_{calc} = 2.545 g cm^{-3}, 10663 reflections measured (6.156° ≤ 2Θ ≤ 71.692°), 1683 unique (R_{int} = 0.0621, R_{sigma} = 0.0388) which were used in all calculations. The final R_1 was 0.0332 for 1398 reflections with $I > 2\sigma(I)$ and wR_2 was 0.0854 for all data.

Supplementary Note 7: Optical microscopy images of sanidine

An image showing the entire surface (Supplementary Fig.12a) was produced by stitching together 20 images at 10x magnification. Details of the surface were imaged with 50x (Supplementary Fig.12b) and 150x (Supplementary Fig. 12c and d) magnification. The overview image in Supplementary Fig. 1a shows a few large step edges which originate from the cleavage. Further, the surface features bright areas, in which interference patterns occur. These patterns are caused by thin sanidine slates due to multiple cleavage. The images with 50x (Supplementary Fig. 12b) and 150x (Supplementary Fig. 12c and d) magnification reveal several straight and parallel steps (approximately along [010]). Steps in different directions are typically curved. Further, the images feature some dark spots.

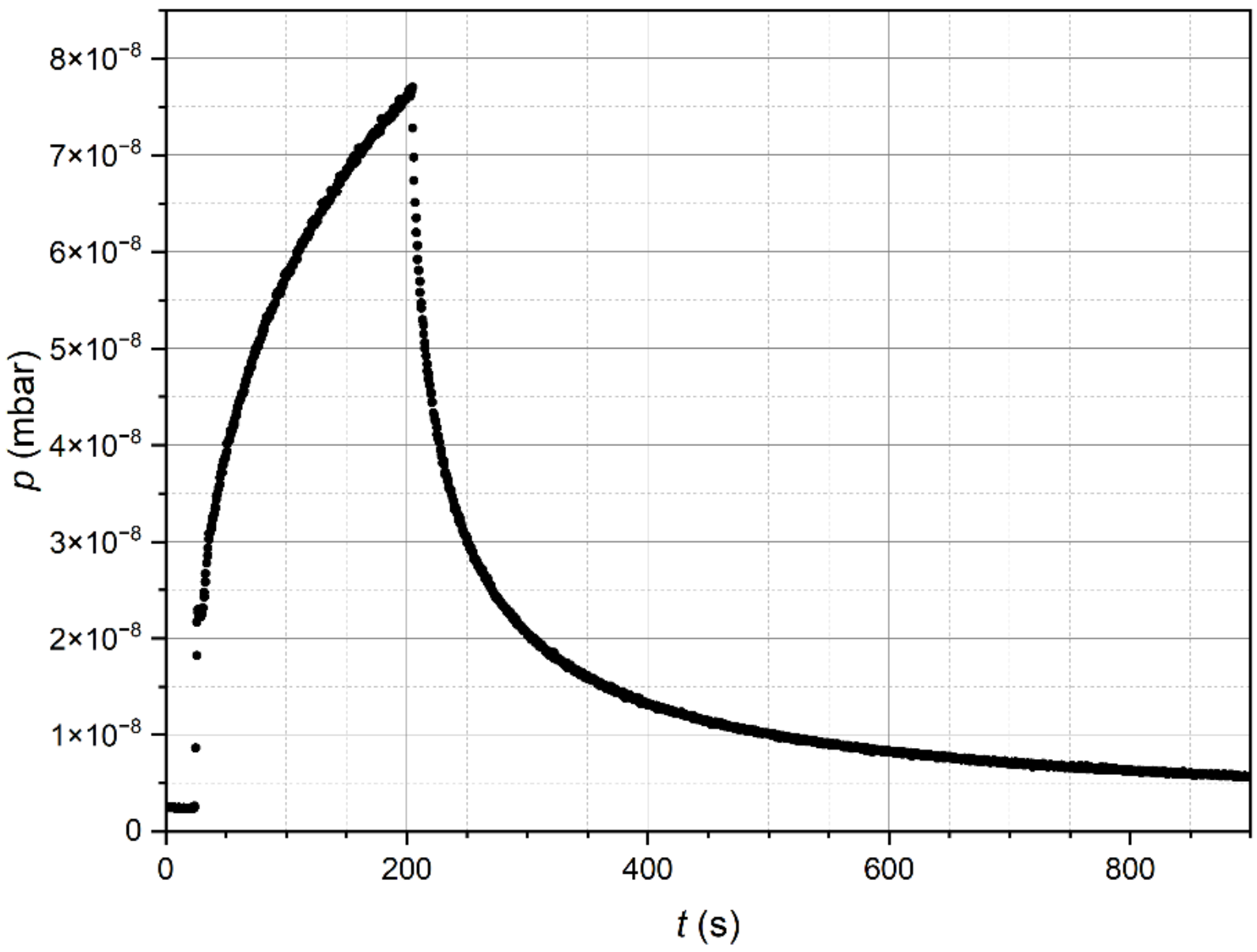

Supplementary Fig. 1 Pressure development while dosing. The water signal intensity measured by a quadrupole mass spectrometer (Hiden HAL 511 3F RC PIC, Hiden Analytical, Great Britain) is plotted against the time. The pressure was calibrated with an ion gauge. Source data are provided as a Source Data file.

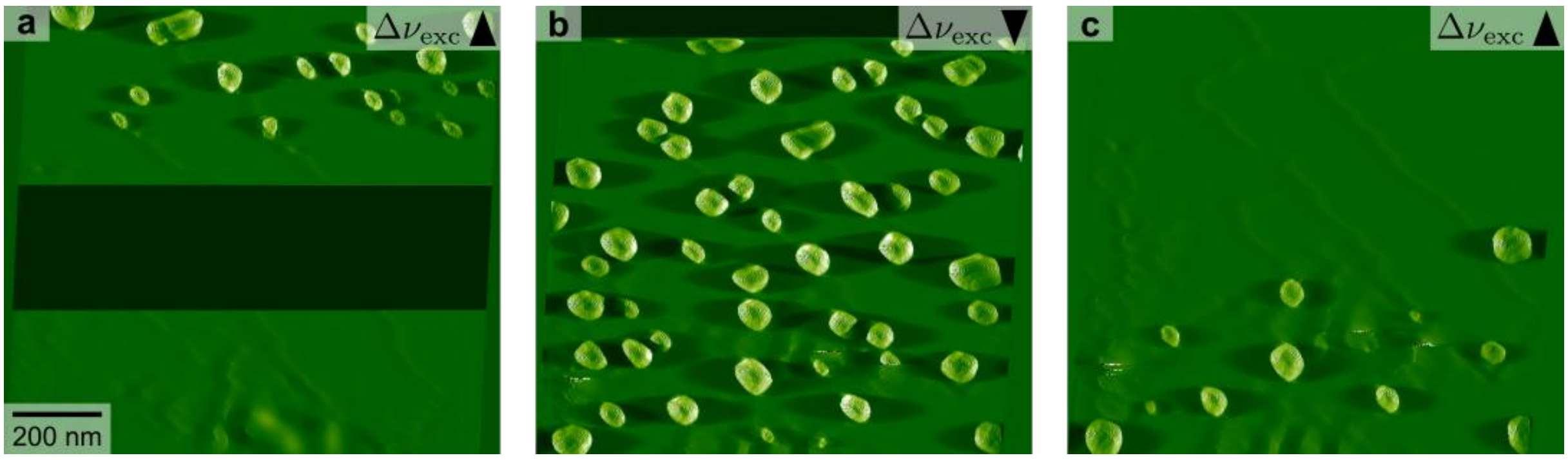

Supplementary Fig. 2 Exemplary ice nucleation and desorption cycle. (**a**) For opening the leak valve, we retract the tip (black area) to arrive at the desired water pressure (here $4.7\cdot10^{-8}$ mbar). Afterwards, imaging is resumed. Ice clusters nucleate and grow while the pressure rises gradually (here to $5.6\cdot10^{-8}$ mbar). (**b**) After closing the leak valve (black area at the top), we image the ice clusters while they shrink. (**c**) After a few minutes all clusters are desorbed. All image are created by adding the trace and retrace image to reveal the cluster shape. The elongated darker areas around each cluster are an artifact due to the limited speed of the feedback loop. The scale bar in (a) applies to all images.

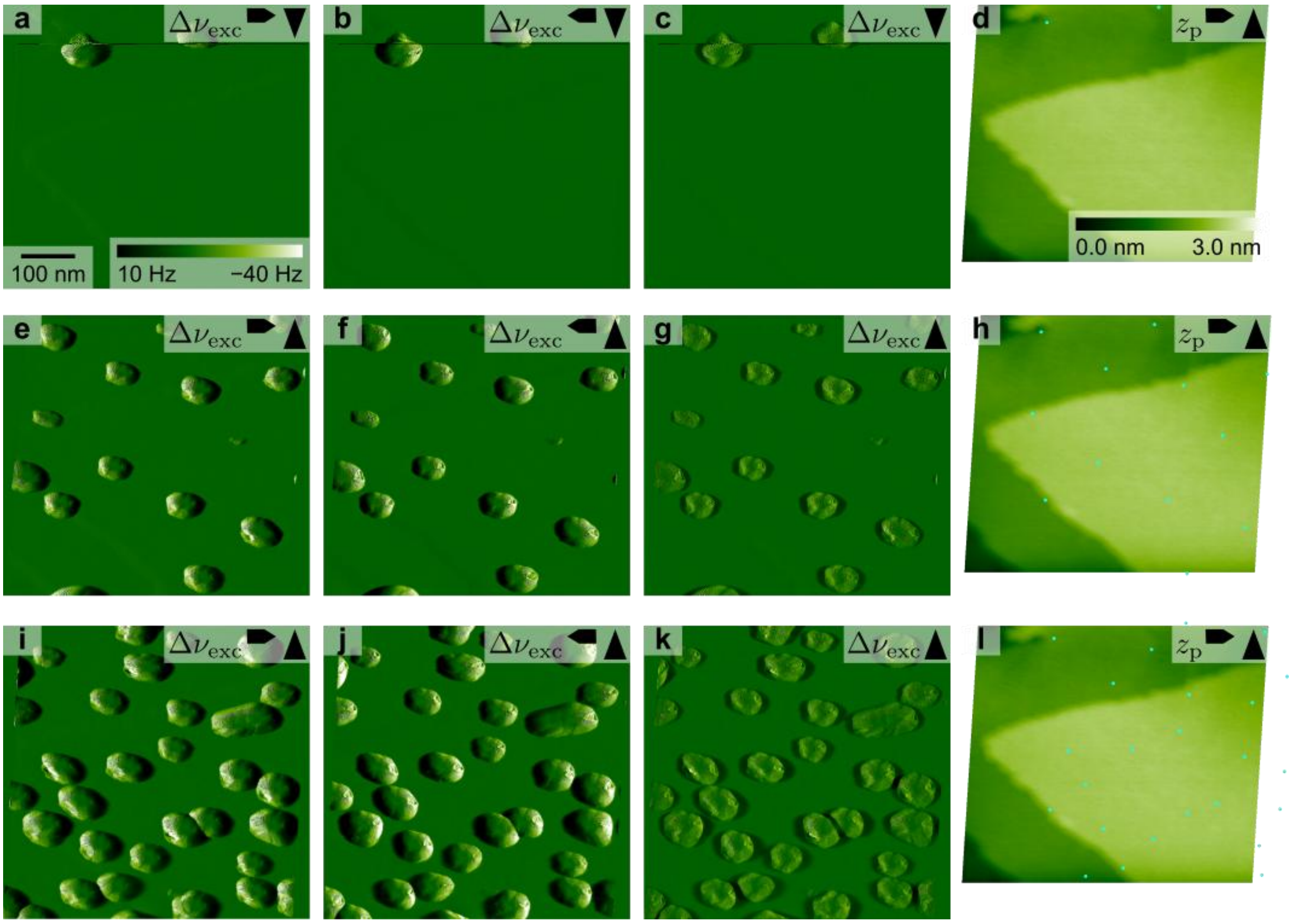

Supplementary Fig. 3 Ice clusters grown in three consecutive nucleation and desorption cycles on microcline. The trace (**a**, **e** and **i**) and retrace (**b**, **f** and **j**) images of three cycles are shown. The color scale in (a) applies to all trace and retrace images. The center of each cluster was determined after adding trace and retrace images (**c**, **g** and **k**) and plotted on top of a topography image (**d**, **h** and **l**). The clusters grew at pressures ranging from $(\mathbf{3.5}-\mathbf{4.6})\cdot\mathbf{10^{-8}}$ mbar (**a**-**d**), $(\mathbf{5.0}-\mathbf{6.8})\cdot\mathbf{10^{-8}}$ mbar (**e**-**h**) and $(\mathbf{5.1}-\mathbf{7.3})\cdot\mathbf{10^{-8}}$ mbar (**i**-**l**). The sample temperature was 145 K. The scale bar in (a) applies to all images. The color scale in (d) applies to all topography images.

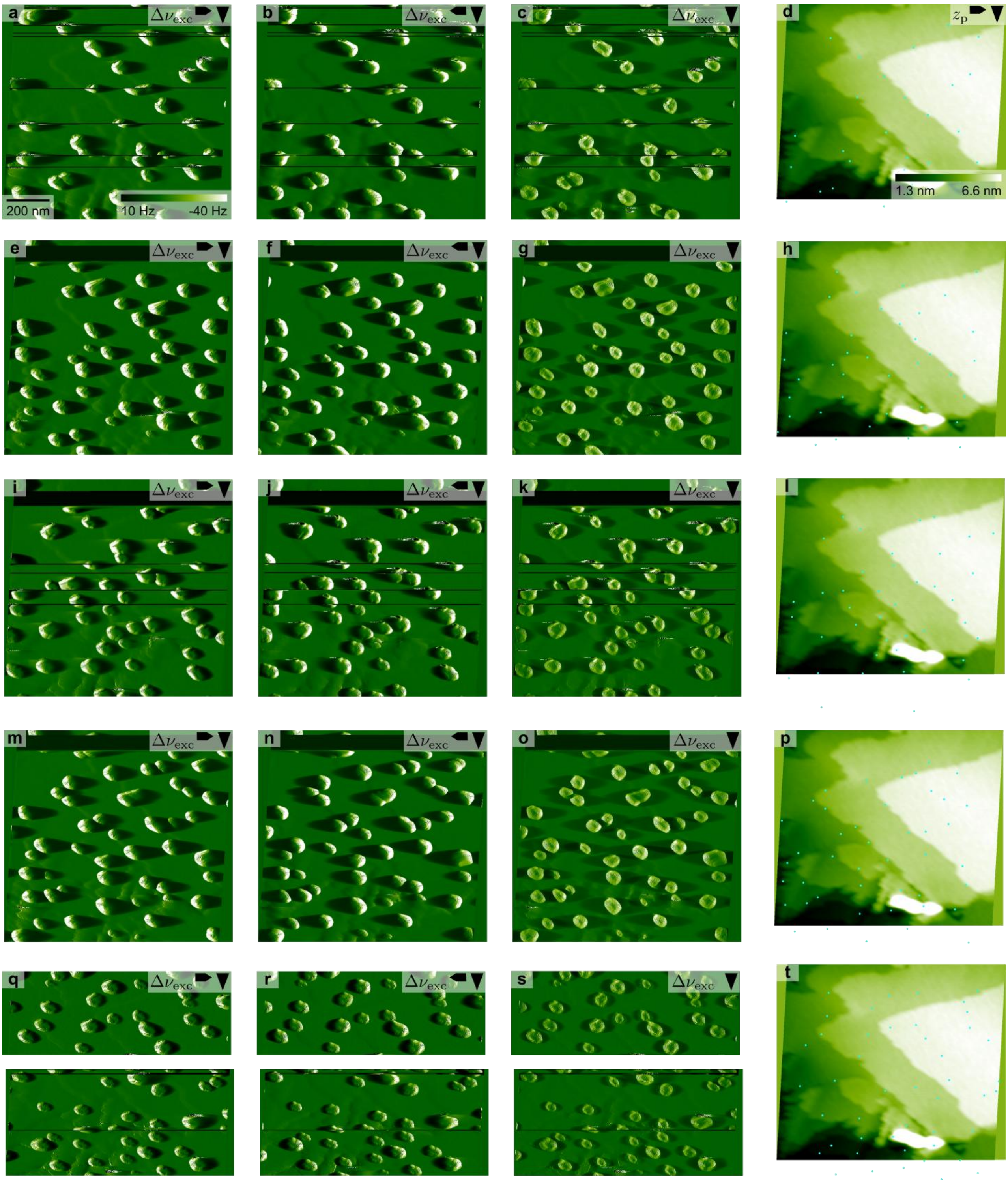

Supplementary Fig. 4 Ice clusters grown in five consecutive nucleation and desorption cycles on microcline. As Supplementary Fig. 3 for five cycles at a larger scale. The clusters grew at pressures ranging from $(\mathbf{2.9-5.3})\cdot\mathbf{10^{-8}}$ mbar (**a-d**), $(\mathbf{3.7-5.6})\cdot\mathbf{10^{-8}}$ mbar (**e-h**), $(\mathbf{4.0-6.0})\cdot\mathbf{10^{-8}}$ mbar (**i-l**) , $(\mathbf{4.7-5.6})\cdot\mathbf{10^{-8}}$ mbar (**m-p**) and $(\mathbf{5.0-8.2})\cdot\mathbf{10^{-8}}$ mbar (**q-t**). The sample temperature was 145 K. The color scale in (a) applies to all trace and retrace images. The scale bar in (a) applies to all images. The color scale in (d) applies to all topography images

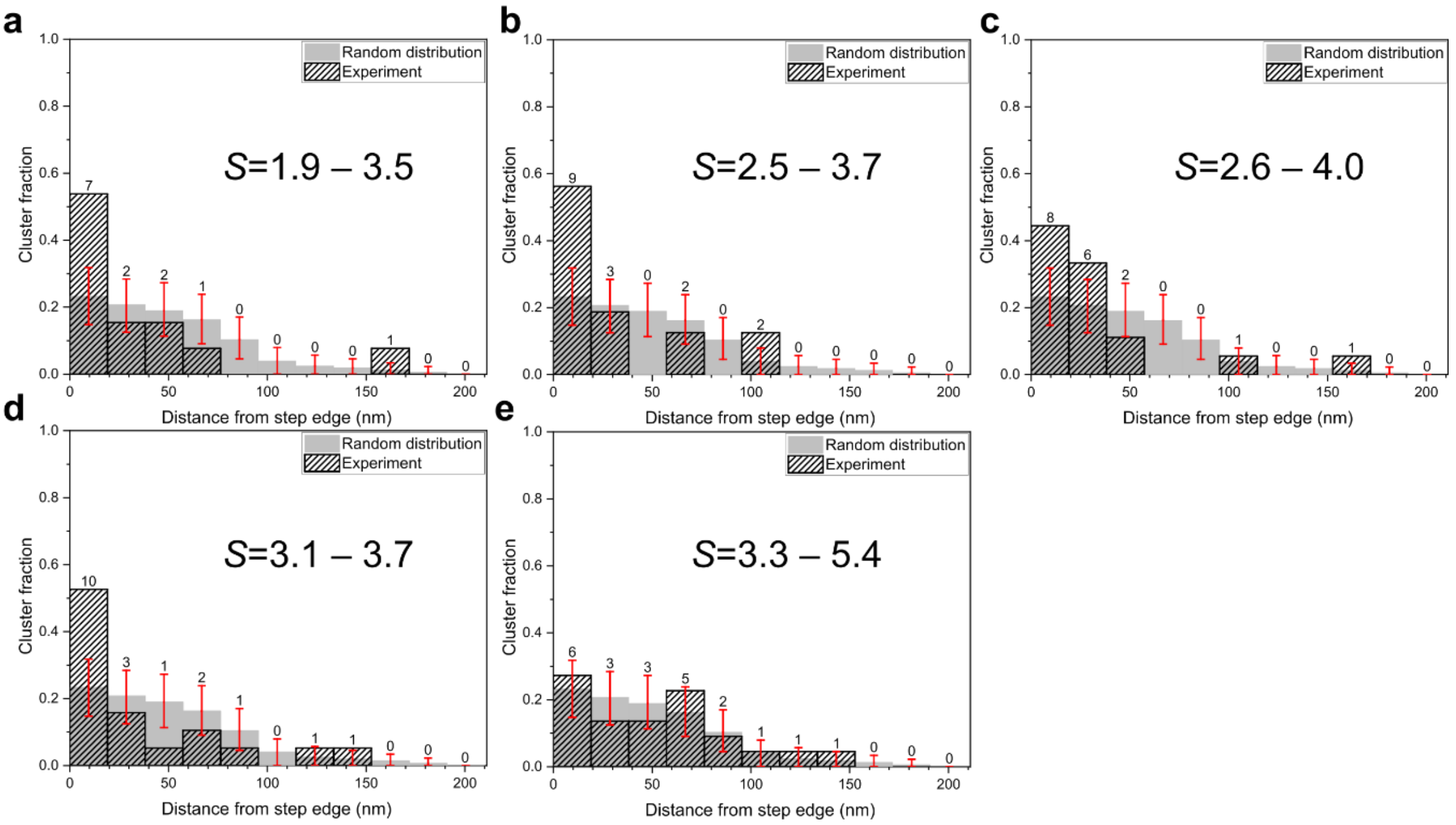

Supplementary Fig. 5 Cluster-step distributions for clusters grown on microcline Distributions of the distance from the nearest step edge of the clusters grown for each individual cycle shown in Supplementary Fig. 5. For comparison, a random distribution is shown by light grey bars. The error bars indicate the 95% credible interval of the random distribution (see Supplementary Note 3). The number of nucleation events per bin is given above each bar (total number of nucleation events: 13 in (a), 16 in (b), 18 in (c), 19 in (d) and 22 in (e)). Source data are provided as a Source Data file.

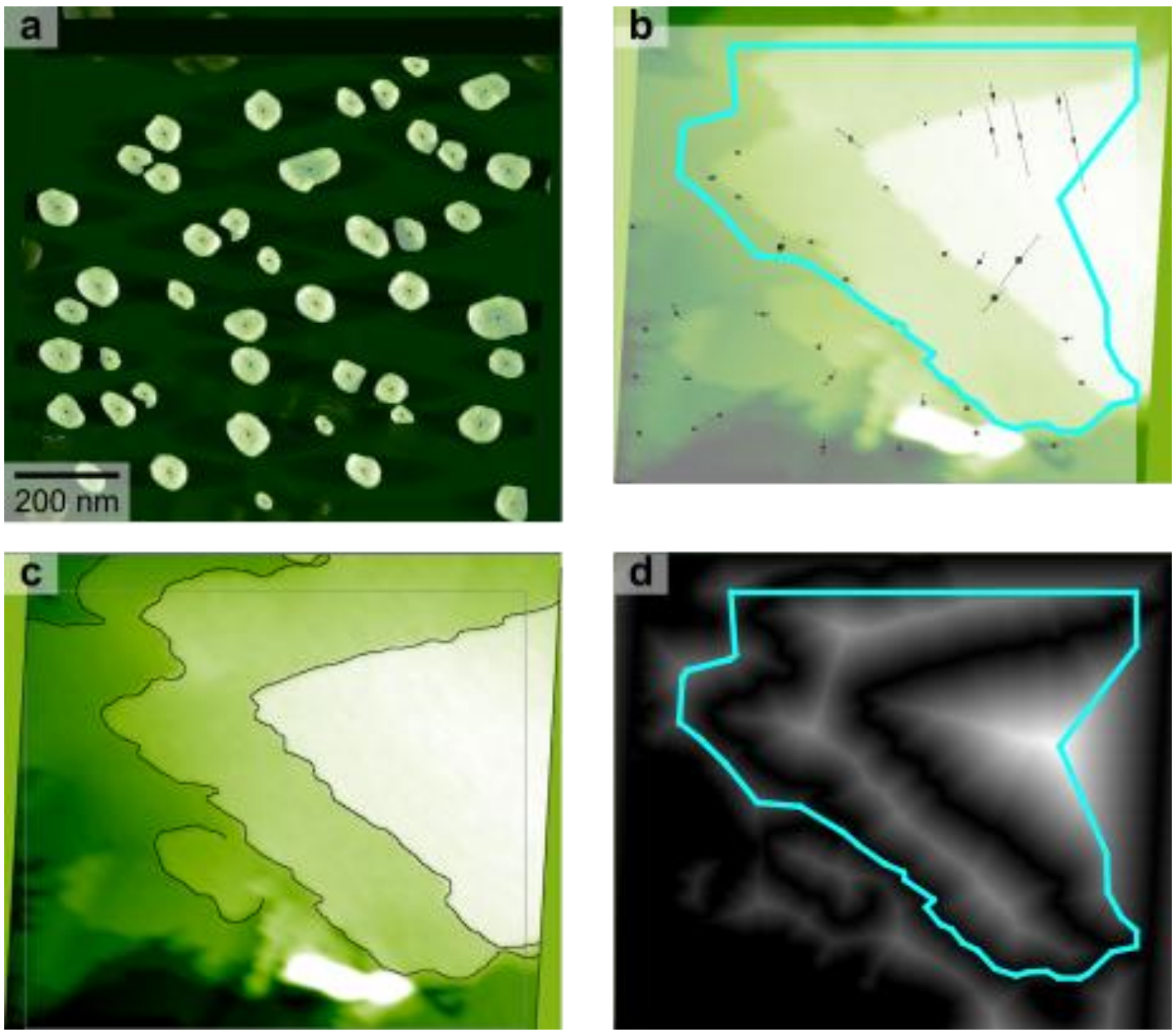

Supplementary Fig. 6 Step-cluster distance measurements. (**a**) AFM image of clusters grown in one cycle with marked centers. (**b**) Distances between each center and the nearest step edge. (**c**) Topography image with marked step edges. The lower image part is excluded from the analysis. (**d**) Grey scale representation of the shortest distance of each pixel from the step edges marked in (c) and the images borders. Exclusively the turquoise area is taken into account for the further analysis as this is area imaged in all consecutive cycles and clusters grown in this area are closer to the marked step edges than to the image's borders or other steps. The scale bar in (a) applies to all images.

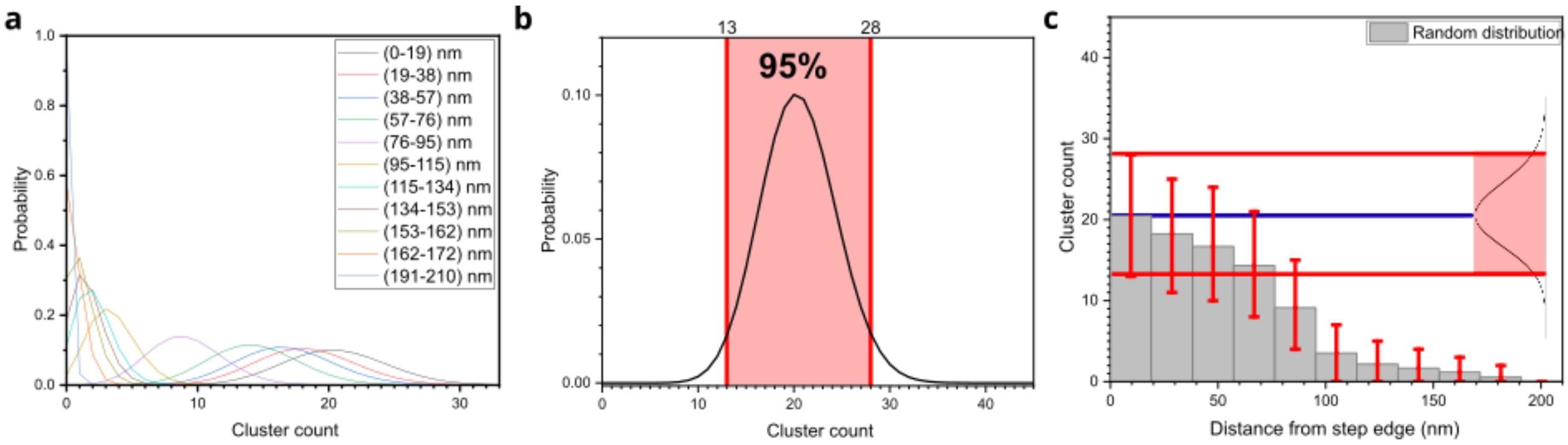

Supplementary Fig. 7 Probability distributions. (**a**) Probability distribution that n cluster nucleate in a specific distance interval from the steps if a random distribution is assumed. (**b**) Probability distribution that *n* clusters nucleate in a distance ranging from 0 to 19 nm from a step edge. The 95% credible interval is marked in red. (**c**) Histogram of the expectation value of observed clusters in a specific distance interval. The error bars mark the 95% credible interval (see Supplementary Note 3). Source data are provided as a Source Data file.

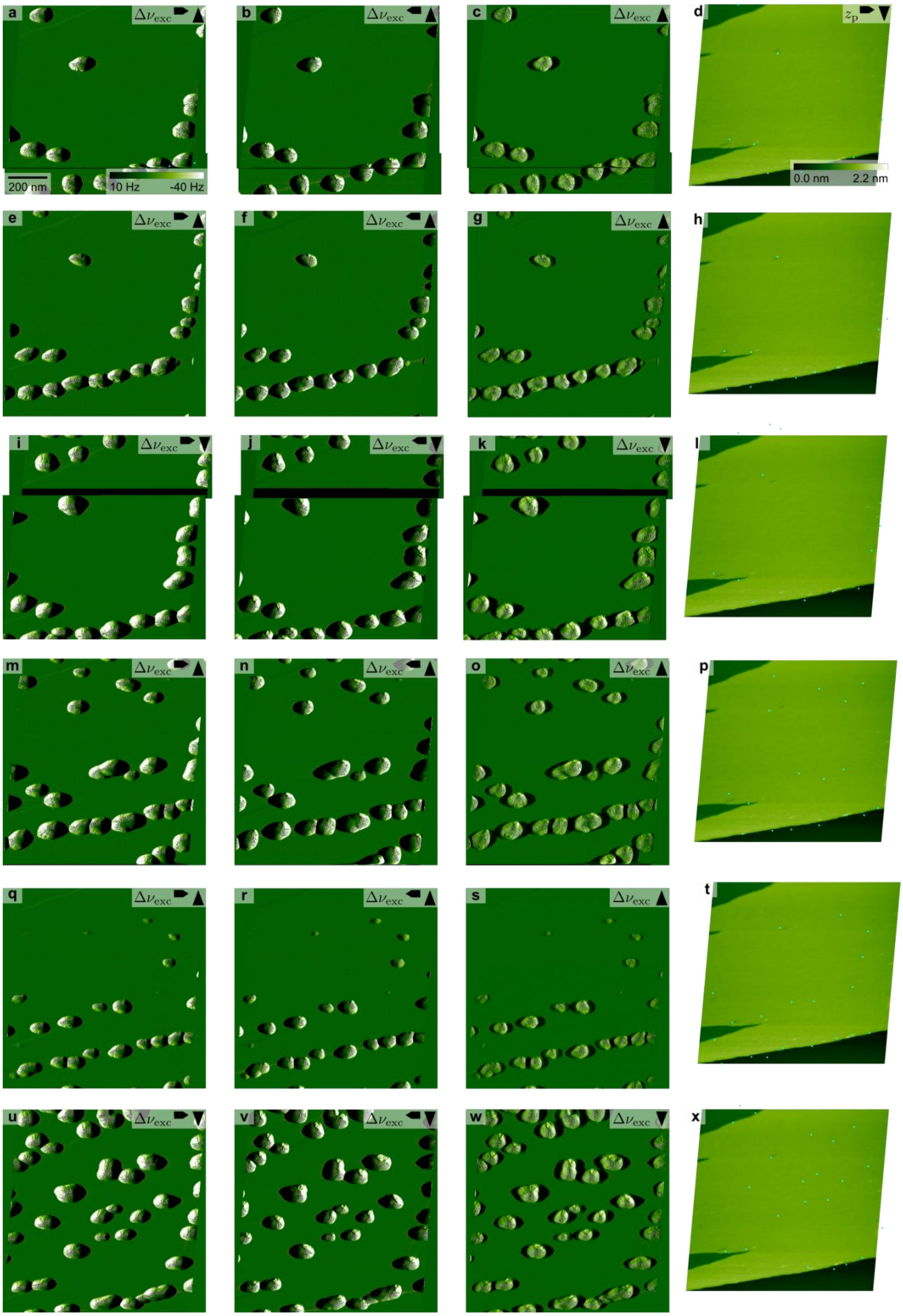

Supplementary Fig. 8 Ice clusters grown in five consecutive nucleation and desorption cycles on sanidine. As Supplementary Fig. 4 for six cycles on sanidine. The clusters grew at pressures ranging from $(\mathbf{5.6-7.9})\cdot\mathbf{10^{-8}}$ mbar (**a-d**), $(\mathbf{6.2-8.4})\cdot\mathbf{10^{-8}}$ mbar (**e-h**), $(\mathbf{6.2-9.3})\cdot\mathbf{10^{-8}}$ mbar(**i-l**), $(\mathbf{6.7-9.8})\cdot\mathbf{10^{-8}}$ mbar (**m-p**), $(\mathbf{7.3-8.7})\cdot\mathbf{10^{-8}}$ mbar (**q-t**) and $(\mathbf{7.3-10})\cdot\mathbf{10^{-8}}$ mbar(**u-x**). The sample temperature was 145 K. The color scale in (a) applies to all trace and retrace images. The scale bar in (a) applies to all images. The color scale in (d) applies to all topography images.

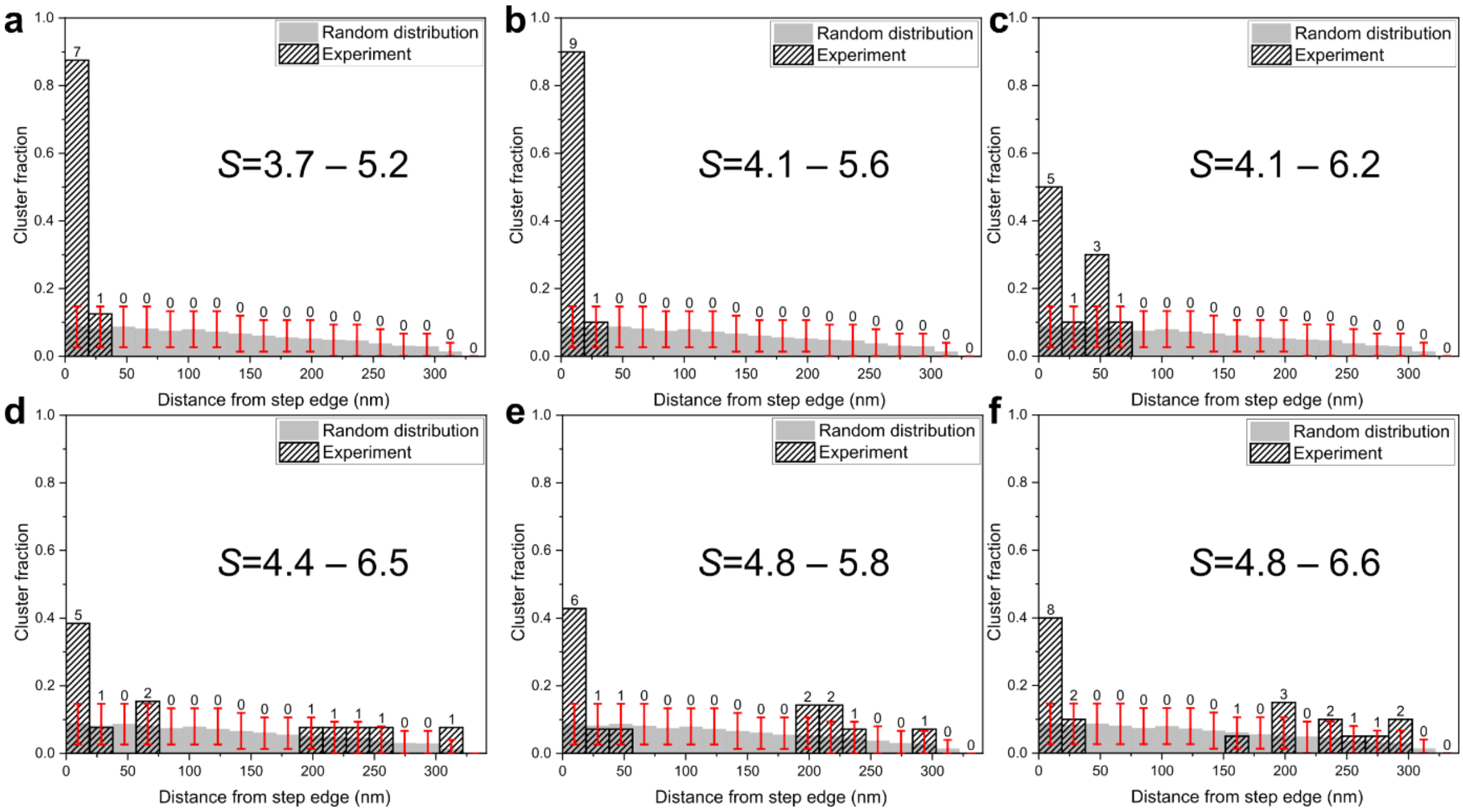

Supplementary Fig. 9 Cluster-step distributions for cluster grown on sanidine As Supplementary Fig. 5 for the cycles on sanidine shown in Supplementary Fig. 8. The number of nucleation events per bin is given above each bar (total number of nucleation events: 8 in (a), 10 in (b), 10 in (c), 13 in (d), 14 in (e) and 20 in (f)). Source data are provided as a Source Data file.

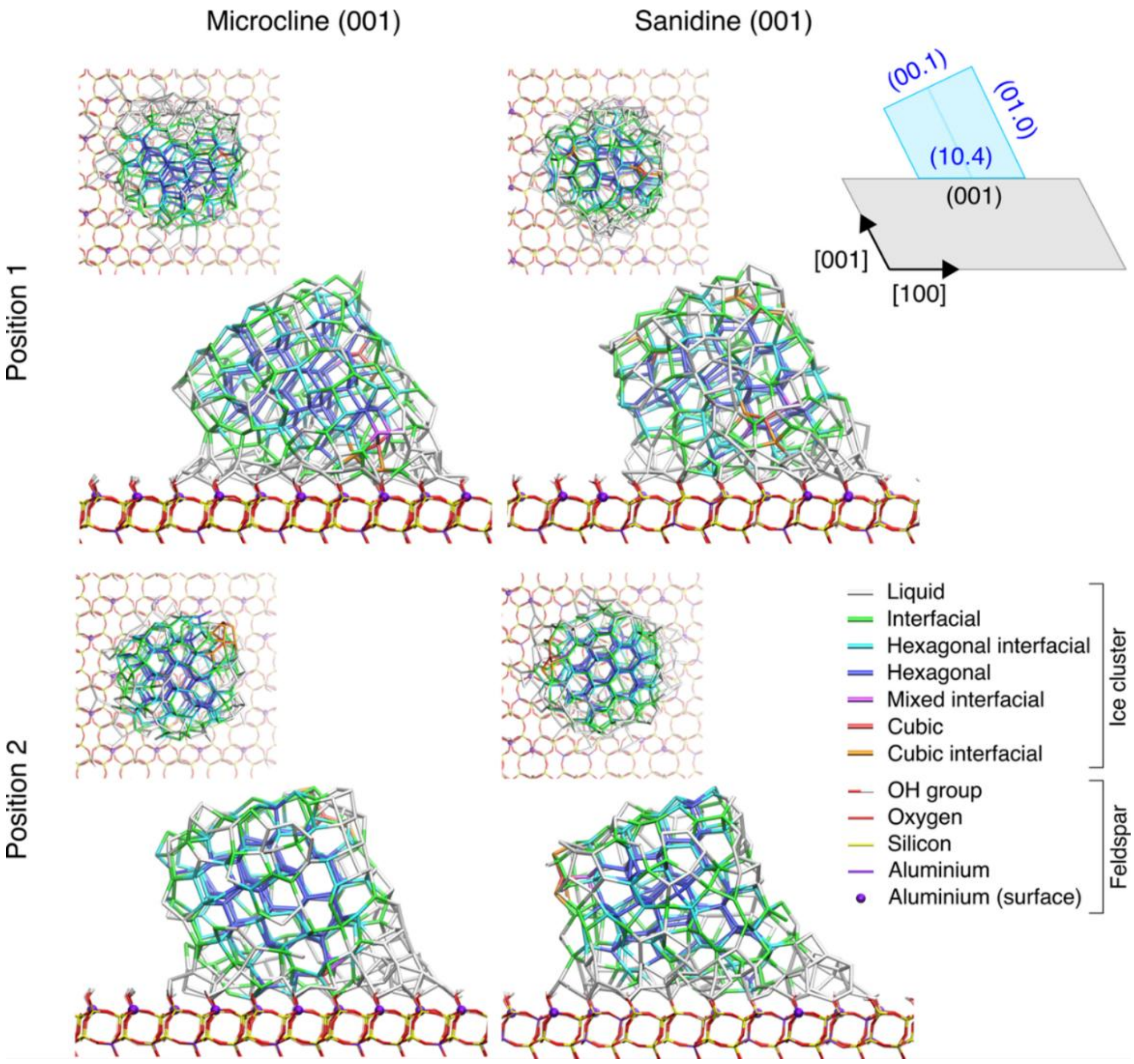

Supplementary Fig. 10 Snapshots of MD simulations at 145 K of hexagonal ice clusters, cut along the (10.4) plane and placed at positions 1 and 2 on microcline and sanidine (001) surfaces. The network of water oxygen atoms within the ice clusters is color-coded according to the local structure determined by the LICH-TEST algorithm[50]. Small insets show the view on the surface along the [001] direction of feldspar.

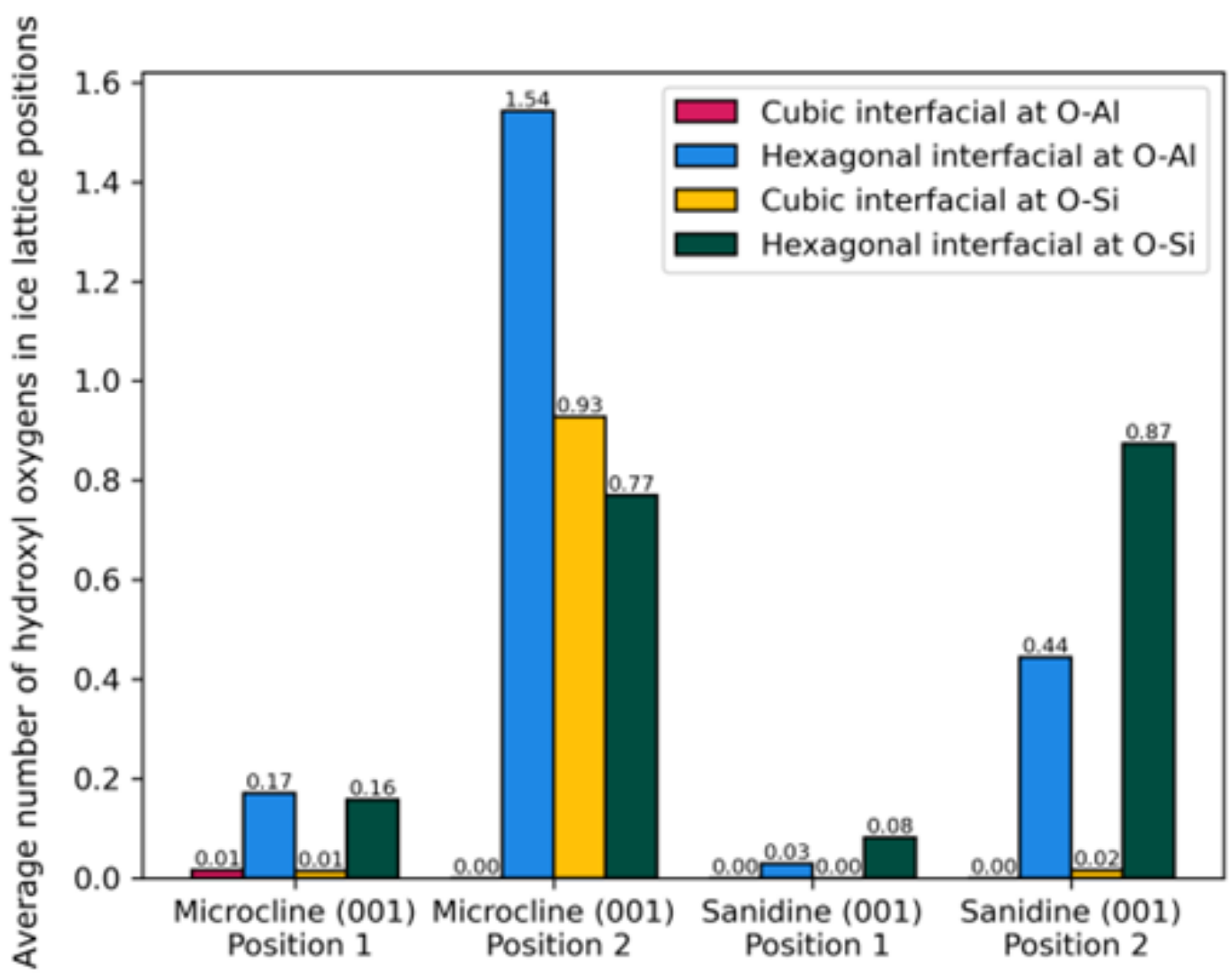

Supplementary Fig. 11 Average number of silanol (O-Si) and aluminol (O-Al) oxygen atoms on microcline and sanidine (001) directly connected to the ice network of the hexagonal ice clusters in positions 1 and 2.

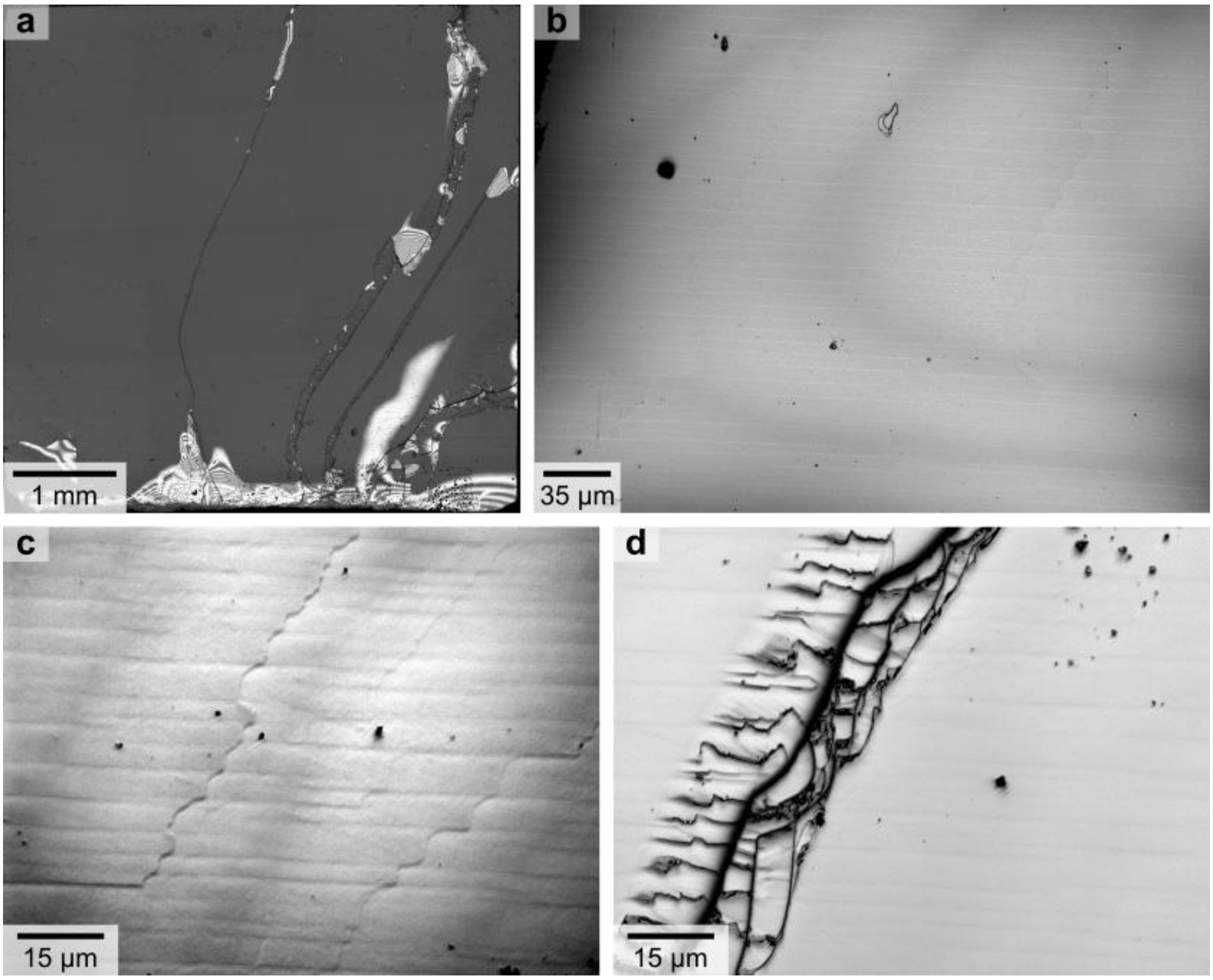

Supplementary Fig. 12 Optical microscopy images taken in air of the sanidine surface cleaved in UHV. The image in **a** was created by stitching 20 images with 10x magnification to capture the whole surface. Further, images with 50x (**b**) and 150x magnification (**c**,**d**) are shown.

Supplementary Table 1 Properties of the parallelograms created by intersecting the unit cell of ice I_h with the [h 0 -h l] plane. Given are the Miller-Bravais indices of the plane, the tilt angle of the plane as well as the dimensions **a** and **b**, and the angle of γ of the intersection parallelogram. Ice planes creating a unit cell matching with the unit cell spanned by the [010] and [310] directions of microcline are marked in red; those matching with the unit cell spanned by the [010] and [410] directions are marked in blue.

Plane	Tilt angle (°)	a (Å)	b (Å)	γ (°)
(1 0 -1 1)	62.0	8.62	4.51	74.8
(1 0 -1 2)	43.2	5.82	4.51	67.2
(1 0 -1 3)	32.1	5.13	4.51	63.9
(1 0 -1 4)	25.2	4.87	4.51	62.4
(1 0 -1 5)	20.6	4.74	4.51	61.6
(1 0 -1 6)	17.4	4.67	4.51	61.1
(1 0 -1 7)	15.0	4.63	4.51	60.9
(1 0 -1 8)	13.2	4.60	4.51	60.7
(1 0 -1 9)	11.8	4.58	4.51	60.5

Supplementary Table 2 Properties of crystal direction on microcline (001). Given are the directional Miller indices, the length of the shortest vector in this direction that is also a linear combination of the primitive lattice vectors of microcline, and the angles with the [100] and [010] direction vectors. To highlight lattice match between ice planes and feldspar directions, the color code is the same as in Supplementary Table 1.

Direction	Length (Å)	Angle with [100] (°)	Angle with [010] (°)
[1 0 0]	8.59	0	87.7
[0 1 0]	12.97	87.7	0
[1 1 0]	7.92	54.9	32.8
[2 1 0]	21.94	36.2	51.5
[3 1 0]	14.66	26.2	61.5
[4 1 0]	37.21	20.4	67.3
[5 1 0]	22.68	16.6	71.1
[1 2 0]	27.65	69.6	18.1
[3 2 0]	37.29	44.0	43.7
[5 2 0]	51.06	30.5	57.2
[7 2 0]	66.44	23.0	64.7

Supplementary Table 3 Comparison of dimensions of microcline (001) and ice I_h intersection parallelograms. The candidate pair with the best lattice match is highlighted in red.

Microcline direction	Ice plane	a_f/a_{ice}	b_f/b_{ice}	$\gamma_f/\gamma_{ice}-1$
[310]	(10-14)	3.01	2.88	1.55%
	(10-15)	3.09	2.88	0.25%
	(10-16)	3.14	2.88	-0.50%
[410]	(10-12)	6.40	2.88	0.19%

Supplementary References

1. Schindelin, J. *et al.* Fiji: an open-source platform for biological-image analysis. *Nat. Methods* **9,** 676–682 (2012).

2. Legland, D., Arganda-Carreras, I. & Andrey, P. MorphoLibJ: integrated library and plugins for mathematical morphology with ImageJ. *Bioinform.* **32,** 3532–3534 (2016).

3. Kiselev, A. *et al.* Active sites in heterogeneous ice nucleation-the example of K-rich feldspars. *Science* **355,** 367–371 (2017).

4. Kuhs, W. F. & Lehmann, M. S. The Structure of Ice-Ih. In *Water Science Reviews 2,* edited by F. Franks (Cambridge University Press1986), pp. 1–66.

5. Löwenstein, W. The Distribution of Aluminum in the Tetrahedra of Silicates and Aluminates. *Am. Mineral.,* 92–96 (1954).

6. Matsumoto, M., Yagasaki, T. & Tanaka, H. GenIce: Hydrogen-Disordered Ice Generator. *J. Comput. Chem.* **39,** 61–64 (2018).

7. Roudsari, G., Veshki, F. G., Reischl, B. & Pakarinen, O. H. Liquid Water and Interfacial, Cubic, and Hexagonal Ice Classification through Eclipsed and Staggered Conformation Template Matching. *J. Phys. Chem. B* **125,** 3909–3917 (2021).

8. Dolomanov, O. V., Bourhis, L. J., Gildea, R. J., Howard, J. A. K. & Puschmann, H. OLEX2 : a complete structure solution, refinement and analysis program. *J. Appl. Crystallogr.* **42,** 339–341 (2009).

9. Sheldrick, G. M. SHELXT - integrated space-group and crystal-structure determination. *Acta Crystallogr. A, Found. Adv.* **71,** 3–8 (2015).

10. Sheldrick, G. M. Crystal structure refinement with SHELXL. *Acta Crystallogr., Sect. C: Struct. Chem.* **71,** 3–8 (2015).
