# Supplementary material for: Ice nucleation on microcline (001) in the absence of active sites
Source: Nat Commun. 2026 Aug 25;17:8652. doi: 10.1038/s41467-026-76548-7 (PMC13507223; doi:10.1038/s41467-026-76548-7)
Supplement: Supplementary file 2 [file 41467_2026_76548_MOESM3_ESM.pdf]

nature portfolio

Corresponding author(s): Florian Schneider, Tobias Dickbreder

Last updated by author(s): Jul 22, 2026

Reporting Summary

Nature Portfolio wishes to improve the reproducibility of the work that we publish. This form provides structure for consistency and transparency in reporting. For further information on Nature Portfolio policies, see our Editorial Policies and the Editorial Policy Checklist.

Statistics

For all statistical analyses, confirm that the following items are present in the figure legend, table legend, main text, or Methods section.

n/a	Confirmed	
☐	☒	The exact sample size (n) for each experimental group/condition, given as a discrete number and unit of measurement
☐	☒	A statement on whether measurements were taken from distinct samples or whether the same sample was measured repeatedly
☒	☐	The statistical test(s) used AND whether they are one- or two-sided<br>*Only common tests should be described solely by name; describe more complex techniques in the Methods section.*
☒	☐	A description of all covariates tested
☒	☐	A description of any assumptions or corrections, such as tests of normality and adjustment for multiple comparisons
☐	☒	A full description of the statistical parameters including central tendency (e.g. means) or other basic estimates (e.g. regression coefficient) AND variation (e.g. standard deviation) or associated estimates of uncertainty (e.g. confidence intervals)
☒	☐	For null hypothesis testing, the test statistic (e.g. F, t, r) with confidence intervals, effect sizes, degrees of freedom and P value noted<br>*Give P values as exact values whenever suitable.*
☒	☐	For Bayesian analysis, information on the choice of priors and Markov chain Monte Carlo settings
☒	☐	For hierarchical and complex designs, identification of the appropriate level for tests and full reporting of outcomes
☒	☐	Estimates of effect sizes (e.g. Cohen's d, Pearson's r), indicating how they were calculated

Our web collection on statistics for biologists contains articles on many of the points above.

Software and code

Policy information about availability of computer code

Data collection	AFM measurements: MATRIX 4.3-V19095587 (V4.3.7) (Omicron, Germany), Mass spectrometry: MASoft (V 11.17.0.1) (Hiden Analytical, UK), MD Simulations: LAMMPS (Paper DOI: 10.1016/j.cpc.2021.108171), GPTA (https://github.com/praiteri/GPTA), LICH-TEST (Paper DOI: 10.1021/acs.jpcb.1c01926), VMD (Paper DOI: 10.1016/0263-7855(96)00018-5)
Data analysis	AFM data analysis: Gwyddion (V2.69, https://gwyddion.net/), unDrift (v1.0, https://doi.org/10.4119/unibi/2984385), ImageJ (V 1.54f)

For manuscripts utilizing custom algorithms or software that are central to the research but not yet described in published literature, software must be made available to editors and reviewers. We strongly encourage code deposition in a community repository (e.g. GitHub). See the Nature Portfolio guidelines for submitting code & software for further information.

Data

Policy information about availability of data

All manuscripts must include a data availability statement. This statement should provide the following information, where applicable:

- Accession codes, unique identifiers, or web links for publicly available datasets
- A description of any restrictions on data availability
- For clinical datasets or third party data, please ensure that the statement adheres to our policy

Unprocessed raw data are provided as Supplementary Data. Source Data are provided with this paper. Further data that supports the findings of this study are available in figshare with the identifier https://doi.org/10.6084/m9.figshare.33061922 . Deposition number 2532369 contains the supplementary crystallographic

data for this paper. These data can be obtained free of charge via the joint Cambridge Crystallographic Data Centre (CCDC) and Fachinformationszentrum Karlsruhe Access Structures service.

Research involving human participants, their data, or biological material

Policy information about studies with human participants or human data. See also policy information about sex, gender (identity/presentation), and sexual orientation and race, ethnicity and racism.

Reporting on sex and gender	This information has not been collected.
Reporting on race, ethnicity, or other socially relevant groupings	This information has not been collected.
Population characteristics	See above.
Recruitment	See above.
Ethics oversight	See above.

Note that full information on the approval of the study protocol must also be provided in the manuscript.

Field-specific reporting

Please select the one below that is the best fit for your research. If you are not sure, read the appropriate sections before making your selection.

☐ Life sciences ☐ Behavioural & social sciences ☒ Ecological, evolutionary & environmental sciences

For a reference copy of the document with all sections, see nature.com/documents/nr-reporting-summary-flat.pdf

Ecological, evolutionary & environmental sciences study design

All studies must disclose on these points even when the disclosure is negative.

Study description	The ice nucleation behaviour on microcline (2 samples, 6 sample positions, 16 cycles) and on sanidine (1 sample, 3 positions, 21 cycles) was investigated unde rulatr-high vacuum conditions at a sample temperature of 145 K (148 K in 8 sanidine cycles) and pressures of the order of 10^(-8) to 10^(-7) mbar.
Research sample	Cuboidal samples cut from microcline and sanidine feldspar (SurfaceNet, Germany, 5 mm x 5 mm x 6 mm).
Sampling strategy	We are investigating the ice nucleation behaviour on flat (001) terraces and small steps of feldspar samples. These surface areas are an intrinsic feature of the observed samples.
Data collection	FS performed the AFm experiments under UHV conditions. BR and RN performed the MD simulations.
Timing and spatial scale	AFm measurements: Microcline crystal 1 (05.06.2025-15.07.2025), Microcline crystal 2 and Sanidine crystal (07.10.2025-27.10.2025).
Data exclusions	We excluded all cycles from the analysis, in which we were not able to image ice crystals and small steps simultaniously or did not observe large terrace areas to obtain a distance histogram (if the terrace is of a size close to the cluster diameter).
Reproducibility	We repeated analoguos experiments on different sample positions and at the same sample position several times. The qualitative observations were the same in every experiment. We were not able to repeat the same high quality of AFM imaging in every experiment as a sharp tip is necessary for this.
Randomization	We did not group our samples.
Blinding	Blinding was not relevant to this study as we do not study any living subjects but minerals. To prevent a bias on our reported qualitative statements we performed distance histograms to obtain objective data.

Did the study involve field work? ☐ Yes ☒ No

Reporting for specific materials, systems and methods

We require information from authors about some types of materials, experimental systems and methods used in many studies. Here, indicate whether each material, system or method listed is relevant to your study. If you are not sure if a list item applies to your research, read the appropriate section before selecting a response.

Materials & experimental systems

n/a	Involved in the study
☒	☐ Antibodies
☒	☐ Eukaryotic cell lines
☒	☐ Palaeontology and archaeology
☒	☐ Animals and other organisms
☒	☐ Clinical data
☒	☐ Dual use research of concern
☒	☐ Plants

Methods

n/a	Involved in the study
☒	☐ ChIP-seq
☒	☐ Flow cytometry
☒	☐ MRI-based neuroimaging

Plants

Seed stocks	We did not investigate any plants.
Novel plant genotypes	We did not investigate any plants.
Authentication	We did not investigate any plants.
