# Supplementary material for: Ice nucleation on microcline (001) in the absence of active sites
Source: Nat Commun. 2026 Aug 25;17:8652. doi: 10.1038/s41467-026-76548-7 (PMC13507223; doi:10.1038/s41467-026-76548-7)
Supplement: Supplementary file 3 [file 41467_2026_76548_MOESM4_ESM.pdf]

nature portfolio

Peer Review File

Ice nucleation on microcline (001) in the absence of active sites

Corresponding Author: Dr Florian Schneider

This file contains all reviewer reports in order by version, followed by all author rebuttals in order by version.

Version 0:

Reviewer comments:

Reviewer #1

(Remarks to the Author)
Review:
Ice nucleation on microcline (001) in the absence of active sites

Summary:

Schneider and colleagues made novel ice nucleation measurements, conducting deposition freezing on surfaces using atomic force microscopy to detect the nanoscale freezing positions relative to surface features. Two samples of feldspar, microcline and sanidine, were tested. Microcline showed clearly that nucleation does not only occur on topographic features such as step-edges but also on the bare surface, showing that active sites are not the sole mechanism by which nucleation on microcline occurs. In the sanidine sample, it is clearly shown that nucleation proceeds predominantly on step-edges. The results presented suggest a strong preference for nucleation on step-edges for sanidine and a weak preference for step-edges on microcline. An explanation for this is provided using molecular dynamics (MD) simulations which showed increased water binding to microcline as a result of greater surface density of aluminol groups.

This paper is of general interest to the ice nucleation community and offers an innovative approach that yields experimental insights into the origin of nucleation. Overall, the research is clearly presented and well communicated. It is suitable for publication in Nature Communication after addressing the following minor revisions.

GENERAL COMMENTS:

The manuscript would benefit from a clearer and possibly more nuanced definition of ‘active sites’. In the current state, active sites are implied to be topography features. However, since the authors convincingly show that microcline nucleates ice also on terraces and demonstrate how the underlying chemistry contributes to this process, it raises the question of whether active sites may be more complex. Are the authors suggesting that atomistic structure-induced nucleation should not be classified as an ‘active site’, or do we need to refine the definition of active sites?

At times too strong of language is used to emphasize the lack of nucleation on active sites on microcline, given that it appears from Figure 3c that ice still preferentially nucleates on active sites. The general claim that ice nucleation appears to be also occuring on the bare atomic surface of (001) microcline, appears well founded.

One aspect that remains underdiscussed in the current manuscript is the saturation dependency component of whether the freezing occurs at either step-edges or terraces. In particular, is there a dependency on the saturation ratio? It is plausible that active sites could still dominate the nucleation on microcline at lower saturation ratios than what is observed in the experiment?

In addition, do the events occur simultaneously at both locations, or does nucleation first occur at one feature and then the other? It would also be interesting to know if the two freezing mechanisms for microcline have different nucleation rates?

SPECIFIC COMMENTS:

Methods:

• Page 12, paragraph 2:
"With XRD, it was confirmed that the samples exhibit a high degree of Al/Si order with 80% of aluminum atoms situated in T1(o) sites." Is it possible that ice nucleation on microcline is supported by atomic scale defect sites that are not resolved by AFM? The evidence provided by the relatively random distribution as well as the MD simulations are compelling in dispelling this notion, but I don't think it can be dismissed outright.

Results & Discussion:

• Figure 2b:
White reference hexagons in Figure 2b could be adjusted to the angle the ice is expected to be growing (shown in figure 2c) for clearer comparison.
• Page 5, paragraph 2:
Finding that "the distance histogram shows no strong preference for distances close to the step edges, indicating that step edges do not significantly enhance the nucleation rate." is not well supported as Figure 3c shows that nucleation is twice as likely to occur on step edges than on the bare surface. Should alter wording to include the fact that the distance histogram does show some preference for nucleation on active sites.
• Page 6, paragraph 2:
Finding that "at the minimal pressure on microcline, ice clusters already nucleate at both the step edges and on the bare terrace." is quite convincing of the broader conclusion that bare surface nucleation on microcline is a significant source of nucleation. This could use elaboration. Is the ratio of observed nucleation events on step edges compared to flat terraces relative to the statistically expected ratio regardless of saturation ratio?

References:

• Reference 44: This is an important caveat that should be stated somewhere in the text of the paper. It introduces the question as to whether at lower saturation ratios, which are more typical of what would be observed under macroscopic conditions, the same trend in the topographic location of ice nucleation would be observed. This is not address in great enough detail. The conclusion would be stronger if this were discussed and this would further motivate the MD simulations.

Appendix:

• Extended data Figures 5 and 6 should be organized in order of increasing water vapour pressure to improve clarity.
• It would potentially be valuable to include distance histograms for extended data Figures 5 and 6. Determining whether the ratio of nucleation events occurring at step-edges is dependent on the water vapor pressure might be of significant value in observing a saturation dependent trend in the preference for nucleation on step edges relative to flat terraces, if such a trend were to exist.

TECHNICAL COMMENTS:

• Page 3, paragraph 1:
"the exceptional role of microcline as ice nucleation at the nanometer scale" should be "the exceptional role of microcline as an ice nuclei at the nanometer scale"

Reviewer #2

(Remarks to the Author)
I co-reviewed this manuscript with one of the reviewers who provided the listed reports. This is part of the Nature Communications initiative to facilitate training in peer review and to provide appropriate recognition for Early Career Researchers who co-review manuscripts.

Reviewer #3

(Remarks to the Author)
This is a very interesting and well-written paper reporting surprising findings regarding the mechanism of ice nucleation on microcline. In contrast to prior work that suggests repeatable occurrence of nucleation on active sites, generally associated with step edges, the authors find that ice nucleation can occur randomly on the bare (001) terrace. MD simulations were further used to confirm hypotheses around the mechanism of nucleation. Contrasting data for a similar mineral that differs only slightly from the structure of microcline are presented to demonstrate how the unique structure of microcline enables its exceptional ice nucleating ability. Overall, this is an excellent study that merits publication, and the results may have important implications for representations of ice nucleation by atmospheric particles, which typically invoke the presence of active sites.

The one aspect that I think merits further discussion by the authors is that their studies were performed at 145 K. This is much colder than the temperatures used in references 26-28 (coldest ~ 233 K). Further, as also mentioned in their footnote (44), the saturation ratios used (2-5) were much higher than in previous studies (1.1-1.3). Is it not possible that different

behavior could be found on the more extreme conditions studied in this work (which are not fully atmospherically relevant)? As Holden et al. (2021) note for their lab studies, “The most active sites appeared to be the least common: typically, we observed a select few very active sites where nucleation occurred on every cycle at relatively low saturation and a much larger number of less active sites that produced crystals at relatively high saturation on one or two cycles only.” That observation from Holden et al. seems to be consistent with the findings from this work: at high supersaturations, the sites where nucleation occurs are more random, whereas the same sites that are active at lower supersaturations continue to show activity; the activation barriers are different. I suggest that the authors consider addressing these potential limitations of their approach.

Reviewer #4

(Remarks to the Author)
In this paper, the authors investigate ice nucleation on microcline (001) using AFM imaging under UHV conditions, with sanidine as a comparison system and MD simulations to rationalize the observations. The key finding is that microcline (001) can promote ice nucleation on flat terraces, rather than at active sites such as steps, cracks, or pores. This is interesting and potentially important, because microcline is known to be unusually efficient among feldspar ice-nucleating particles. The combination of repeated deposition/desorption AFM experiments, comparison with sanidine, and atomistic modeling makes the work promising.

The main concern is statistical robustness. The work shows five cycles on microcline and three on sanidine in the main text. The authors should explicitly state how many independent samples, cleaves, surface regions, and nucleation events were analyzed for both microcline and sanidine. It would be helpful to show whether the near-random distribution of nucleation positions on microcline holds for individual cycles or independent regions, rather than only in pooled data.

While the MD simulations provide useful support, they should be interpreted more cautiously. The simulations place preformed ice clusters on the surfaces and compare their stability, but they do not directly observe nucleation or calculate a nucleation barrier (which obviously is very challenging by itself). Thus, the simulations do not necessarily prove that this is the unique or operative nucleation pathway.

The broader atmospheric relevance should also be stated carefully. The UHV experiments at 145 K and relatively high saturation ratios are valuable for nanoscale mechanistic imaging, but they are not direct demonstrations of atmospheric immersion or deposition freezing conditions.

Overall, I think this is a technically interesting and potentially significant study. With more careful wording and a more cautious interpretation of the simulations, the manuscript could become suitable for publication in Nature Communications.

Version 1:

Reviewer comments:

Reviewer #1

(Remarks to the Author)
Schneider and colleagues have substantially improved their manuscript and have incorporated our suggestions well. My co-reviewer and I therefore recommend this paper for publication.

Reviewer #2

(Remarks to the Author)
I co-reviewed this manuscript with one of the reviewers who provided the listed reports. This is part of the Nature Communications initiative to facilitate training in peer review and to provide appropriate recognition for Early Career Researchers who co-review manuscripts.

Reviewer #3

(Remarks to the Author)
The authors have done an excellent job in responding to the comments of the reviewers. I have no further suggestions.

Reviewer #4

(Remarks to the Author)
The authors have provided more details about nucleation experiments in SI. I agree that the data set supports the claim that ice nucleation is near random on microcline. They also revised the interpretation of MD simulation and addressed the limitation of the experiments in the context of atmospheric relevance. I believe the current form of the manuscript is acceptable for Nature Communications.

Open Access This Peer Review File is licensed under a Creative Commons Attribution 4.0 International License, which permits use, sharing, adaptation, distribution and reproduction in any medium or format, as long as you give appropriate credit to the original author(s) and the source, provide a link to the Creative Commons license, and indicate if changes were made.
In cases where reviewers are anonymous, credit should be given to 'Anonymous Referee' and the source.
The images or other third party material in this Peer Review File are included in the article’s Creative Commons license, unless indicated otherwise in a credit line to the material. If material is not included in the article’s Creative Commons license and your intended use is not permitted by statutory regulation or exceeds the permitted use, you will need to obtain permission directly from the copyright holder.
To view a copy of this license, visit https://creativecommons.org/licenses/by/4.0/

Point-by-point response

Note: We indicate the reviewer's comments by black text, our answers are given in blue text, and new text in the revised manuscript is given in green text.

Reviewer #1:
Schneider and colleagues made novel ice nucleation measurements, conducting deposition freezing on surfaces using atomic force microscopy to detect the nanoscale freezing positions relative to surface features. Two samples of feldspar, microcline and sanidine, were tested. Microcline showed clearly that nucleation does not only occur on topographic features such as step-edges but also on the bare surface, showing that active sites are not the sole mechanism by which nucleation on microcline occurs. In the sanidine sample, it is clearly shown that nucleation proceeds predominantly on step-edges. The results presented suggest a strong preference for nucleation on step-edges for sanidine and a weak preference for step-edges on microcline. An explanation for this is provided using molecular dynamics (MD) simulations which showed increased water binding to microcline as a result of greater surface density of aluminol groups.

This paper is of general interest to the ice nucleation community and offers an innovative approach that yields experimental insights into the origin of nucleation. Overall, the research is clearly presented and well communicated. It is suitable for publication in Nature Communication after addressing the following minor revisions.

> We thank the Reviewer for the concise summary of our main findings and the positive evaluation of our work.

1.1: The manuscript would benefit from a clearer and possibly more nuanced definition of 'active sites'. In the current state, active sites are implied to be topography features. However, since the authors convincingly show that microcline nucleates ice also on terraces and demonstrate how the underlying chemistry contributes to this process, it raises the question of whether active sites may be more complex. Are the authors suggesting that atomistic structure-induced nucleation should not be classified as an 'active site', or do we need to refine the definition of active sites?

> Triggered by this insightful comment, we now clearly state the two common, but deviating definitions of active sites in literature. On page 2 of our manuscript, we now write:
> "These sites are known to be rare as only few of these sites are identified in large-scale microscope images[28]. We note that the term "active site" is used in two different ways in the literature. First, each position that nucleates ice is termed an active site. Within this definition, the number of active sites strongly increases with the ice saturation ratio[31]. Second, only rare and distinct positions, which nucleate ice at low saturation ratios are termed active sites[28]. This notion implies that ice nucleation occurs preferentially at the same positions, i.e., the active sites, in subsequent experiments."
> In our manuscript we use the second definition, which commonly associates active sites with rare topographic features.

As flat terraces are not rare, but frequent features of crystals, we would not classify them as active sites. We cannot rule out the presence of atomic defects, in fact, many atomic defects are expected. However, if these defect sites act as the main nucleation sites on the terraces, we would still not classify them as active sites as they are an intrinsic and frequent feature of the surface. In summary, as nucleation is observed at many different sites, instead of a few distinct ones, we would not assign these as active sites.

1.2: At times too strong of language is used to emphasize the lack of nucleation on active sites on microcline, given that it appears from Figure 3c that ice still preferentially nucleates on active sites. The general claim that ice nucleation appears to be also occuring on the bare atomic surface of (001) microcline, appears well founded.

From this comment we learn that the main claim of our manuscript, the observation of ice nucleation on the bare terrace, was clear. However, some of our sentences appeared to be too strong, given that nucleation at step edges is, indeed, also observed. We did not intend to negate the preference of ice nucleation at step edges but highlight the fact that ice nucleation does also occur on the bare terrace. Triggered by this Reviewer comment, we carefully evaluated the entire manuscript and identified sentences that could suggest the wrong claim that ice nucleation would be limited to the bare terraces. We rephrased potentially misleading sentences, e.g., on page 3 we now write "nucleation positions **on the terrace** scatter in a random fashion, providing no evidence for preferential nucleation at active sites" and microcline's "ice nucleation activity **is not limited** to active sites but also manifests itself on the bare terrace".

1.3: One aspect that remains underdiscussed in the current manuscript is the saturation dependency component of whether the freezing occurs at either step-edges or terraces. In particular, is there a dependency on the saturation ratio? It is plausible that active sites could still dominate the nucleation on microcline at lower saturation ratios than what is observed in the experiment? In addition, do the events occur simultaneously at both locations, or does nucleation first occur at one feature and then the other?

As we aim to investigate atomic scale mechanisms, we are inherently limited to investigating a small surface area. To observe ice nucleation on a small area, larger saturation ratios are needed compared to nucleation experiments investigating large surfaces areas, e.g., with optical microscopy. We understand the request to investigate the saturation ratio dependency, however, the experimentally accessible range is rather narrow, as we do not observe any ice nucleation at small saturation ratios during reasonable observational time scales (several minutes up to half an hour). Even at the high supersaturations used, the nucleation of clusters typically takes a few minutes, see Supplementary Fig. 3. Nevertheless, from the distance distributions of individual nucleation cycles recorded at different saturation ratios (shown in Supplementary Fig.11 and 12), we are able to observe a weak dependency on the saturation ratio: while at lower saturation ratios nucleation is favored at steps, this preference is not observed for the highest saturation ratio on microcline. Based on this finding, we can conclude that active sites play a more dominant role with decreasing supersaturation. Still, the initial clusters that nucleate after opening the

leak valve, are observed both at the step edges and on the bare terrace as shown in Supplementary Fig. 3.

We thank the Reviewer for raising this interesting question, which is now discussed with an additional analysis added in the Supplementary Information (Figs. 11 and 12) and on page 6 of the main text: "To confirm whether this near-random distribution of nucleation positions also holds for individual cycles, we also show the histograms of each individual cycle in Supplementary Fig. 11. As the saturation ratio in each cycle differs, comparing these histograms reveals information regarding the saturation ratio dependence of the observed ice nucleation: while we observe a preference for the nucleation at step edges at the lower observed saturation ratios, the distance distribution at the highest saturation ratio perfectly matches a random distribution. Nevertheless, at all investigated saturation ratios, we observed ice nucleation at step edges as well as on the bare terrace."

1.4: It would also be interesting to know if the two freezing mechanisms for microcline have different nucleation rates?

We agree with the Reviewer that the nucleation rates for both freezing mechanisms are interesting. As we observe a slight preference for nucleation at step edges, both nucleation mechanisms are expected to have different overall rates. However, the nucleation rate on terraces should be dependent on surface area, while the nucleation rate at step edges is expected to depend on their length and orientation. While we think that this is an interesting question, we feel that a systematic investigation of the nucleation rates is beyond the scope of the current manuscript. Further, as the imaging time of one AFM image is on the order of minutes, AFM measurements are not suitable to determine nucleation rates accurately.

1.5: Methods:

• Page 12, paragraph 2:

"With XRD, it was confirmed that the samples exhibit a high degree of Al/Si order with 80% of aluminum atoms situated in T1(o) sites." Is it possible that ice nucleation on microcline is supported by atomic scale defect sites that are not resolved by AFM? The evidence provided by the relatively random distribution as well as the MD simulations are compelling in dispelling this notion, but I don't think it can be dismissed outright.

As we outlined in our answer to point 1.1, we cannot dispel entirely the notion of atomic scale defect sites supporting ice nucleation on microcline. The Al/Si order of 80 % indicates that 20 % of silicon and aluminum atoms are swapped. Hence, on average every fifth unit cell contains a point defect. However, we are certain that these defects do not act as active sites. First, they are a frequent surface feature, which does not classify as a rare active site. This state this explicitly in our manuscript: "We cannot rule out the presence of atomic defects, which could be responsible for the ice nucleation. In fact, many atomic defects are expected to exist on the surface. Nevertheless, if these defects were to act as nucleation sites, we would not classify them as active sites as these defects are an intrinsic and frequent feature of the surface." Second, if a lower Al-Si order would enhance the ice nucleating efficacy of feldspar minerals, sanidine would be more efficient in ice

nucleation compared to microcline. This contrasts with our and previous findings. To summarize, while we cannot exclude that ice nucleation is supported by some rare point defects, we can exclude support by the frequent swapping of Al and Si atoms.

Results & Discussion:

1.6: Figure 2b:
White reference hexagons in Figure 2b could be adjusted to the angle the ice is expected to be growing (shown in figure 2c) for clearer comparison.

We thank for this constructive correction and adjusted the hexagonal shape and orientation to reflect the expected shape based on the findings of Kiselev et al. for a clearer comparison.

1.7: Page 5, paragraph 2:
Finding that "the distance histogram shows no strong preference for distances close to the step edges, indicating that step edges do not significantly enhance the nucleation rate." is not well supported as Figure 3c shows that nucleation is twice as likely to occur on step edges than on the bare surface. Should alter wording to include the fact that the distance histogram does show some preference for nucleation on active sites.

We agree with the Reviewer and now state on pages 5-6:
"Directly at the step edge the nucleation probability is higher than what is expected for a random distribution, indicating that the step edge does induce nucleation. However, we also observe significant nucleation events at large distances from the step edge. This finding demonstrates that ice nucleation also occurs on the bare terrace."

1.8: Page 6, paragraph 2:
Finding that "at the minimal pressure on microcline, ice clusters already nucleate at both the step edges and on the bare terrace." is quite convincing of the broader conclusion that bare surface nucleation on microcline is a significant source of nucleation. This could use elaboration. Is the ratio of observed nucleation events on step edges compared to flat terraces relative to the statistically expected ratio regardless of saturation ratio?

As correctly noted by the Reviewer, the main conclusion is that the bare surface of microcline represents a significant source of nucleation.
We understand the interest in evaluating the saturation ratio dependency of our findings. A clear statement regarding the saturation ratio dependency is difficult as the pressure continuously rises during dosing (see Supplementary Fig. 2). However, as requested we have analyzed the freezing cycles individually and provide the corresponding histograms for microcline in Supplementary Fig. 11 and for sanidine in Supplementary Fig. 12. As already indicated in Fig. 3c, we find a slight preference for nucleation at the step edge in case of microcline for low saturation ratios. For higher saturation ratios a distribution close to the random distribution is achieved. In contrast, on sanidine nucleation at step edges prevails for the entire saturation ratio range investigated. We have added a discussion of this interesting aspect on page 6 of the main text:

"To confirm whether this near-random distribution of nucleation positions also holds for individual cycles, we also show the histograms of each individual cycle in Supplementary Fig. 11. As the saturation ratio in each cycle differs, comparing these histograms reveals information regarding the saturation ratio dependence of the observed ice nucleation: while we observe a preference for the nucleation at step edges at the lower observed saturation ratios, the distance distribution at the highest saturation ratio perfectly matches a random distribution. Nevertheless, at all investigated saturation ratios, we observed ice nucleation at step edges as well as on the bare terrace."

We also added a discussion in the Supplementary Information: "The comparison of the distributions obtained for different saturation ratios reveals that at most investigated saturation ratios the nucleation rate directly at the step edges is higher than expected for a random distribution by a factor of about two. However, we also observe ice nucleation on the bare terrace. At the largest observed supersaturation, the experimentally observed distribution does not deviate from a random distribution.

In contrast, on sanidine, the experimentally observed distribution strongly deviates from a random distribution at all investigated saturation ratios. At lower saturation ratios, which are higher than the saturation ratios on microcline, we observe ice nucleation exclusively at step edges. At higher saturation ratios, we observe a bimodal distribution: ice nucleation is predominantly observed directly at the step edges and, to a lesser degree, at distances larger than 100 nm from the step edges, but never in between. This bimodal distribution indicates that the nucleation occurs predominantly at step edges resulting in a lower water concentration in the vicinity of the step edges. As a consequence, the decreased water concentration reduces the probability of further nucleation events close to the step edges. These findings indicate that the nucleation rate on sanidine on the bare terrace is much smaller than the nucleation rate at steps."

References:

1.9: Reference 44: This is an important caveat that should be stated somewhere in the text of the paper. It introduces the question as to whether at lower saturation ratios, which are more typical of what would be observed under macroscopic conditions, the same trend in the topographic location of ice nucleation would be observed. This is not address in great enough detail. The conclusion would be stronger if this were discussed and this would further motivate the MD simulations.

Following the Reviewer's suggestion we now state the important information from the former reference 44 in the main text of the manuscript on page 4:
„ We are aware that the reported saturation ratios are significantly higher than typically reported saturation ratios in macroscopic ice nucleation experiments. This difference is because of the fact that we only image a small fraction of the surface. Consequently, we need higher saturation ratios to observe ice nucleation in the limited image frame on the timescale of minutes. "

Further, we now add a comprehensive discussion section on pages 9-10, in which this important fact is also addressed: "Nanoscale imaging inherently implies that only a small fraction of the surface can be imaged. Therefore, higher saturation rations are

needed as compared to, e.g., light microscopy studies that cover a macroscopically large surface area." Still the obtained results provide "a crucial missing link between mesoscale experimental observations and molecular-level theoretical modeling. […] Our results demonstrate unambiguously that even the bare (001) surfaces of microcline and sanidine, despite being structurally very similar, exhibit significantly different ice nucleation efficacies." Regarding the dependence on the saturation ratio we refer to our reply to point 1.8.

Appendix:

1.10: Extended data Figures 5 and 6 should be organized in order of increasing water vapour pressure to improve clarity.

For improved clarity and better comparison, we have reorganized these Figures as requested.

1.11: It would potentially be valuable to include distance histograms for extended data Figures 5 and 6. Determining whether the ratio of nucleation events occurring at step-edges is dependent on the water vapor pressure might be of significant value in observing a saturation dependent trend in the preference for nucleation on step edges relative to flat terraces, if such a trend were to exist.

We agree with this comment and refer to our answer to comments 1.3 and 1.8.

TECHNICAL COMMENTS:

1.12: Page 3, paragraph 1:
"the exceptional role of microcline as ice nucleation at the nanometer scale" should be "the exceptional role of microcline as an ice nuclei at the nanometer scale"

We thank the Reviewer for this hint und corrected the corresponding sentence (using singular instead of plural): "Our work, thus, underscores the exceptional role of microcline as an ice nucleator at the nanometer scale by showing that its ice nucleation activity is not limited to active sites but also manifests itself on the bare terrace."

Reviewer #2:
I co-reviewed this manuscript with one of the reviewers who provided the listed reports. This is part of the Nature Communications initiative to facilitate training in peer review and to provide appropriate recognition for Early Career Researchers who co-review manuscripts.

We thank the Reviewer for his/her evaluation of our work.

Reviewer #3:
This is a very interesting and well-written paper reporting surprising findings regarding the mechanism of ice nucleation on microcline. In contrast to prior work that suggests repeatable occurrence of nucleation on active sites, generally associated with step edges, the authors find that ice nucleation can occur randomly on the bare (001) terrace. MD

simulations were further used to confirm hypotheses around the mechanism of nucleation. Contrasting data for a similar mineral that differs only slightly from the structure of microcline are presented to demonstrate how the unique structure of microcline enables its exceptional ice nucleating ability. Overall, this is an excellent study that merits publication, and the results may have important implications for representations of ice nucleation by atmospheric particles, which typically invoke the presence of active sites.

> We are very delighted to read that the Reviewer refers to our work as "an excellent study that merits publication".

3.1: The one aspect that I think merits further discussion by the authors is that their studies were performed at 145 K. This is much colder than the temperatures used in references 26-28 (coldest ~ 233 K). Further, as also mentioned in their footnote (44), the saturation ratios used (2-5) were much higher than in previous studies (1.1-1.3). Is it not possible that different behavior could be found on the more extreme conditions studied in this work (which are not fully atmospherically relevant)? As Holden et al. (2021) note for their lab studies, "The most active sites appeared to be the least common: typically, we observed a select few very active sites where nucleation occurred on every cycle at relatively low saturation and a much larger number of less active sites that produced crystals at relatively high saturation on one or two cycles only." That observation from Holden et al. seems to be consistent with the findings from this work: at high supersaturations, the sites where nucleation occurs are more random, whereas the same sites that are active at lower supersaturations continue to show activity; the activation barriers are different. I suggest that the authors consider addressing these potential limitations of their approach.

> We agree that the implications of our low-temperature and high saturation ratio experiments were not sufficiently discussed in the previous version of the manuscript. As stated in our answer to point 1.9 above, we now explicitly discuss footnote 44 in the main text on page 4. Additionally, we have expanded the discussion of the saturation ratio dependence as outlined in our reply to point 1.8. Further, motivated by the Reviewer's comparison of our data with the study of Holden et al., we included a discussion section in the manuscript, in which we relate our experimental conditions to environmental conditions and previous studies such as the one from Holden et al. (2021). Within this discussion section, we now highlight that our findings have two important implications:
>
> "We further note that on both microcline and sanidine the preference for nucleation at step edges increases with decreasing saturation ratio. This finding is fully consistent with previous optical microscopy experiments showing that, at low saturation ratios, ice nucleation is restricted to a small number of active sites, whereas at higher saturation ratios it occurs at a larger number of more randomly scattered positions[29].
>
> Our findings do have important implications: First, they show that even minor atomic-scale variations can translate into substantial differences in the ice nucleation efficacy. Thus, the different ice nucleating behavior of two closely related minerals, such as microcline and sanidine, may be induced by only subtle structural differences. For microcline and sanidine, this different ice nucleation behavior can most likely be assigned to the higher aluminol density on microcline. Second, as discussed above, active sites such as pores are composed of facets such as the (001) surface investigated

here. It has previously been demonstrated that ice nucleation in pores is strongly enhanced by the inverse Kelvin effect, which promotes water condensation even below water saturation, followed by freezing[49]. If the atomic scale structure would be irrelevant under such conditions, the ice crystals emerging from these active sites would not be expected to exhibit any preferred orientation. This is, however, in contrast to experiments on the mesoscale clearly showing well-oriented ice crystals growing repeatedly at the same active site[30]. Taken together, these observations indicate that atmospheric ice nucleation cannot be understood in terms of mesoscale topography or molecular-scale structure alone. Rather, a combination of both effects has to be considered, as the topographic enhancement associated with the inverse Kelvin effect and the molecular-level influence of crystallographic match are likely to act cooperatively."

Reviewer #4:

In this paper, the authors investigate ice nucleation on microcline (001) using AFM imaging under UHV conditions, with sanidine as a comparison system and MD simulations to rationalize the observations. The key finding is that microcline (001) can promote ice nucleation on flat terraces, rather than at active sites such as steps, cracks, or pores. This is interesting and potentially important, because microcline is known to be unusually efficient among feldspar ice-nucleating particles. The combination of repeated deposition/desorption AFM experiments, comparison with sanidine, and atomistic modeling makes the work promising.

We thank the Reviewer for the careful analysis of our main findings and the overall positive assessment of our work being potentially important and promising.

4.1: The main concern is statistical robustness. The work shows five cycles on microcline and three on sanidine in the main text. The authors should explicitly state how many independent samples, cleaves, surface regions, and nucleation events were analyzed for both microcline and sanidine. It would be helpful to show whether the near-random distribution of nucleation positions on microcline holds for individual cycles or independent regions, rather than only in pooled data.

We understand the concern regarding statistical robustness. To address this concern, we now explicitly present the requested information in the Supplementary Information on page 5: "We performed ice nucleation experiments on two microcline and one sanidine crystal. Each crystal was cleaved only once as thin as possible within our sample holder to minimize the deviation between surface temperature and the temperature measured by the thermocouple attached to the sample holder.

In total, we performed 16 nucleation and desorption cycles at six different sample sites on microcline. In each cycle we observed that ice nucleation is not limited to step edges but occurs also on the bare surface.

On sanidine we performed 13 nucleation and desorption cycles at two different sample positions. (In another experiment we performed another eight cycles at one sample position. However, the sample temperature was about 3 K higher than in the other experiment, resulting in a higher pressure ($p > 1 \cdot 10^{-7}$ mbar) necessary to induce ice nucleation. This is why this series was neglected for this analysis.) In all these experiments, we observed ice nucleation predominantly at step edges. Ice nucleation

at the bare terrace was exclusively observed for the highest supersaturations. We want to note that the imaged shape of the crystals is sensitive to the quality of the used AFM tip, often resulting in a broad and blurred mapping of the grown ice crystals. Further, the simultaneous imaging of both the ice crystals and surface features such as step edges, is experimentally challenging and requires a sharp tip. Nevertheless, this simultaneous imaging is necessary to determine the distance between the center of each ice crystal and the closest step edge. Moreover, to compare the difference in nucleation between bare terrace areas and step edges, we need AFM images showing also large terrace areas. For images showing only areas with small terrace widths, the corresponding distance distribution would be limited to only small distances. Hence, we carefully chose only those cycles that provide simultaneous imaging of both well-defined ice crystals and large terrace areas, which were five out 16 for microcline and six out of 13 for sanidine." and on page 14: "In total, we evaluated 88 (75) nucleation events on microcline (sanidine)."

We are convinced that this data set is convincing and entirely sufficient to proof the qualitative conclusions stated in our manuscript. In particular, in all experiments (even with data that was not suitable for a precise determination of distance distributions) we observed that ice nucleation was never limited to step edges on microcline, but always occurs simultaneously at the bare terrace, while on sanidine ice nucleation occurs predominantly at step edges in all cycles.

To confirm that the near-random distribution not only holds for the pooled data, but also individual experiments, we determined the distance histograms for each analyzed cycle and and now show these separatly as Supplementary Fig. 11 and 12. These histograms of individual cycles also clearly reveal that ice nucleation is not limited to step edges on microcline but always also occurs on the bare terrace, resulting in a near-random distribution, with about twice as many ice clusters than expected directly at the step edge. We want to note that at the highest observed saturation ratio the histogram is almost perfectly described by a random distribution with no preference for step edges.

4.2: While the MD simulations provide useful support, they should be interpreted more cautiously. The simulations place preformed ice clusters on the surfaces and compare their stability, but they do not directly observe nucleation or calculate a nucleation barrier (which obviously is very challenging by itself). Thus, the simulations do not necessarily prove that this is the unique or operative nucleation pathway.

We agree with the Reviewer that the MD simulations have to be interpreted more cautiously. Hence, we adopted our interpretation in the manuscript on page 8: "Thus, our simulations support the proposed mechanism of stacking ice Ih with its (10.4) surface on feldspar (001), which explains the orientation of the ice crystals in the experimental AFM images in Fig. 2. While these simulations do not reveal the atomistic ice nucleation mechanism directly, the differences in ice binding observed are in agreement with the experimental evidence presented above that ice nucleation on microcline (001) occurs more readily than on sanidine (001)."

4.3: The broader atmospheric relevance should also be stated carefully. The UHV experiments at 145 K and relatively high saturation ratios are valuable for nanoscale

mechanistic imaging, but they are not direct demonstrations of atmospheric immersion or deposition freezing conditions.

> This comment is related to the concerns raised by Reviewer 1 (point 1.9) and Reviewer 3 (point 3.1) and we also refer to our answers to the above comments. We acknowledge the necessity to clearly state both the limitations and advantages of our approach. Thus, we now state on page 9 in our discussion that our experiments are intrinsically limited to small surface areas, featuring flat terraces and small step edges: "Nanoscale imaging inherently implies that only a small fraction of the surface can be imaged. Therefore, higher saturation rations are needed as compared to, e.g., light microscopy studies that cover a macroscopically large surface area. Thus, the conditions used in the present experiments are inherently different from those that are typically present in the atmosphere. Accordingly, the obtained results should not be interpreted as a direct representation of atmospheric ice nucleation. Rather, they should be viewed as a mechanistic framework that complements studies performed under atmospherically more relevant conditions. Such studies have consistently demonstrated that ice nucleation is dominated by active sites, which can be associated with mesoscale topographic features, such as cracks and pores[27,28,30]. However, while these approaches identify the importance of topographic features, they are not able to provide insights into the underlying, nanoscopic processes that govern ice nucleation at the molecular level. In contrast, theoretical studies typically address ice nucleation at the molecular scale, but often lack direct experimental verification[22,24,25]. In this context, the present study provides a crucial missing link between mesoscale experimental observations and molecular-level theoretical modeling. Because high-resolution imaging within surface features such as cracks and pores is currently not feasible, we are restricted to less active surfaces regions, such as flat terraces and small step edges. Nevertheless, mesoscale surface features are composed of different facets on the nanoscale. In particular, because the (001) surface of microcline is the most stable one, it is plausible that this surface is also exposed within crack and pore structures. Our results demonstrate unambiguously that even the bare (001) surfaces of microcline and sanidine, despite being structurally very similar, exhibit significantly different ice nucleation efficacies. On microcline (001), ice nucleation is observed already at a minimal pressure of $4.6\cdot10^{-8}$ mbar and occurs almost randomly scattered across the surface with only a small preference for step edges at the investigated conditions. In contrast, on sanidine (001), a substantially higher pressure is necessary to induce ice nucleation, which occurs predominantly at step edges and only marginally on the bare terrace."

4.4: Overall, I think this is a technically interesting and potentially significant study. With more careful wording and a more cautious interpretation of the simulations, the manuscript could become suitable for publication in Nature Communications.

> We thank the Reviewer for appreciating our new technological approach. Regarding the wording, we want to refer to our answer to Reviewer 1 (comment 1.2). Further, we are convinced that the changes to the manuscript, in particular the new discussion section, which puts our work into a broader context and highlights both

limitations and opportunities, greatly enhanced the value of our contribution to the scientific community.

Editorial requests:

- The abstract has been shortened and references have been removed. The final sentence now begins with “Our findings provide …”
- All information that was previously included in the Extended Data are now either moved to the new Methods section or the Supplementary Information.
- The data availability statement now reads: “The raw-data files corresponding to the data presented in the manuscript are given in the Supplementary Data file. All, further data are available from the corresponding authors (fschneider8@uni-bielefeld.de; tobias.dickbreder@univie.ac.at) upon reasonable request.”
